# Aggregation-induced emission photosensitizer-based photodynamic therapy in cancer: from chemical to clinical

**DOI:** 10.1186/s12951-022-01553-z

**Published:** 2022-07-26

**Authors:** Zijuan Meng, Huiying Xue, Tingting Wang, Biao Chen, Xiyuan Dong, Lili Yang, Jun Dai, Xiaoding Lou, Fan Xia

**Affiliations:** 1 State Key Laboratory of Biogeology and Environmental Geology, Faculty of Materials Science and Chemistry, China University of Geosciences, Wuhan, 430074 China; 2 Institute of Pathology, Tongji Hospital, Tongji Medical College, Huazhong University of Science and Technology, Wuhan, 430034 China; 3 Department of Obstetrics and Gynecology, Tongji Hospital, Tongji Medical College, Huazhong University of Science and Technology, Wuhan, 430034 China

**Keywords:** Aggregation-induced emission, Photosensitizers, Photodynamic therapy, Cancer therapy, Clinical translation

## Abstract

Cancer remains a serious threat to human health owing to the lack of effective treatments. Photodynamic therapy (PDT) has emerged as a promising non-invasive cancer treatment that consists of three main elements: photosensitizers (PSs), light and oxygen. However, some traditional PSs are prone to aggregation-caused quenching (ACQ), leading to reduced reactive oxygen species (ROS) generation capacity. Aggregation-induced emission (AIE)-PSs, due to their distorted structure, suppress the strong molecular interactions, making them more photosensitive in the aggregated state instead. Activated by light, they can efficiently produce ROS and induce cell death. PS is one of the core factors of efficient PDT, so proceeding from the design and preparation of AIE-PSs, including how to manipulate the electron donor (D) and receptor (A) in the PSs configuration, introduce heavy atoms or metal complexes, design of Type I AIE-PSs, polymerization-enhanced photosensitization and nano-engineering approaches. Then, the preclinical experiments of AIE-PSs in treating different types of tumors, such as ovarian cancer, cervical cancer, lung cancer, breast cancer, and its great potential clinical applications are discussed. In addition, some perspectives on the further development of AIE-PSs are presented. This review hopes to stimulate the interest of researchers in different fields such as chemistry, materials science, biology, and medicine, and promote the clinical translation of AIE-PSs.

**Graphical Abstract:**

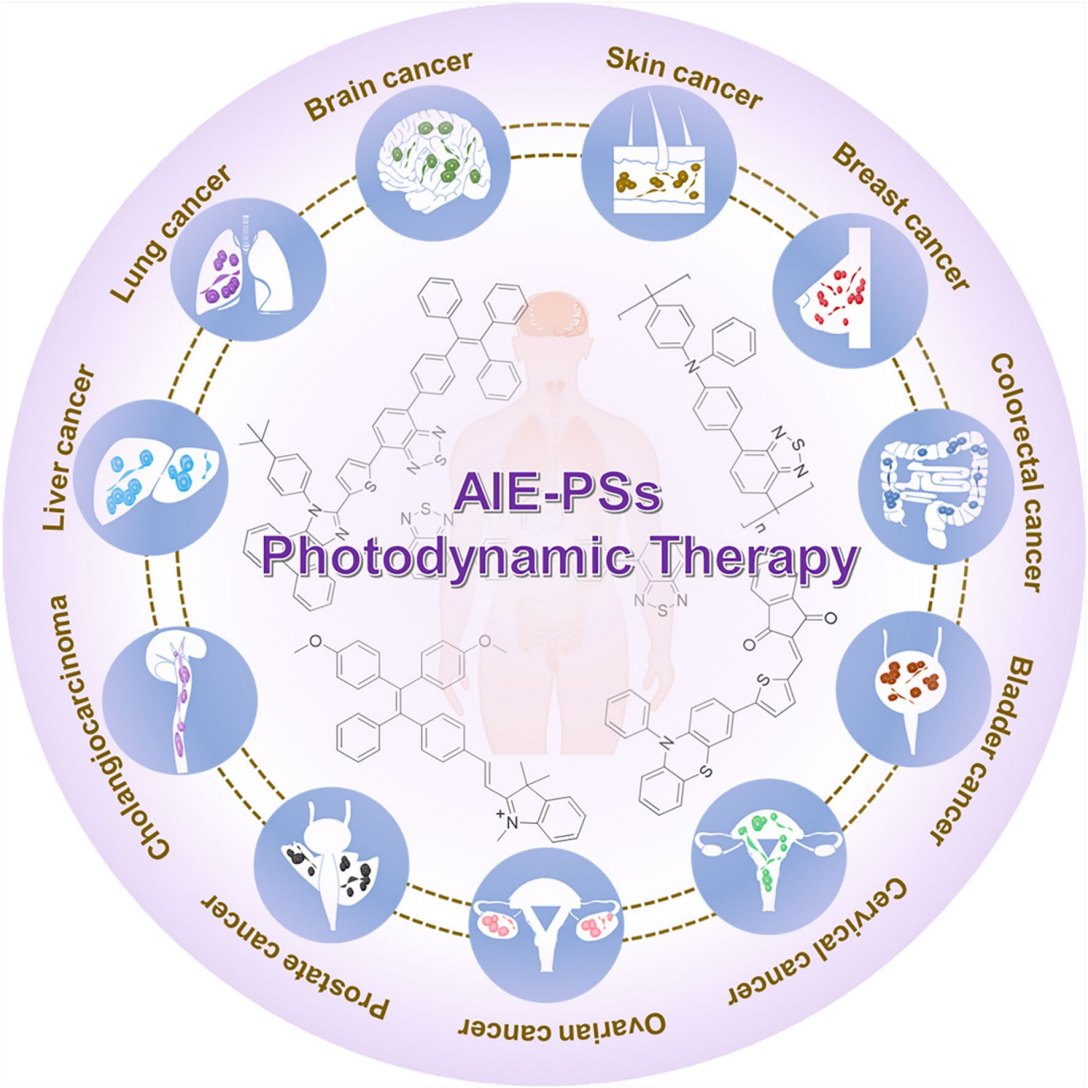

## Background

The incidence and mortality rates of cancer are increasing rapidly in countries around the world [1, 2]. Cancer is a mortal disease that is relatively difficult to cure because of its uncontrollable and ultrafast proliferation properties [3, 4]. Thus, it is of profound significance for human health to explore highly effective and active therapeutic approaches for cancer. With the advent of PDT, approaches for treating tumors have been further expanded [5–8]. PDT has undergone a long period of development since the specific cytotoxic effects of combining acridine dyes with light to lead to tumor ablation were demonstrated in 1900 [9]. Due to its minimal invasiveness, high safety, in situ maneuverability and high spatiotemporal precision, it has eventually been used as a photo-regulated therapeutic modality for cancers and other diseases in clinical therapy [10–12]. Although PDT was the first drug-device combination authorized by the United States Food and Drug Administration (FDA), it is still not fully utilized in clinical practice [13, 14]. Therefore, improving the utilization rate and therapeutic ability of PDT is an urgent need to advance clinical tumor treatment.

During PDT, PSs are initiated by light irradiation at a specific wavelength, which matches the absorption of PSs, to produce ROS, primarily including ^1^O_2_, O^2•−^, •OH and peroxides, to damage the pathological lesions [15]. However, the practical application shows that ROS has a short half-life (less than 40 ns) and a limited diffusion distance (up to 20 nm) in the organism, which is also a limitation that traditional PDT is not yet widely used for clinical tumor therapy [16]. So far, several PSs have been approved for clinical treatment of tumors, such as porfimer sodium (Photofrin) (HPD) [17], 5-aminolevulinic acid (ALA) [18], ALA esters [19], temoporfin (Foscan) (mTHPC) [20, 21] and verteporfin [22, 23]. Unfortunately, the photosensitivity of conventional PSs is severely impaired by ACQ and photobleaching, resulting in limited application in tumor therapy [24–27]. It was not until 2001 that Tang's research group discovered a phenomenon opposite to ACQ, which showed that the higher the degree of aggregation of fluorescent molecules, the stronger the fluorescence, and named this phenomenon as AIE [28]. In the aggregated state, the fluorescence emission of AIE luminogens (AIEgens) is enhanced, as is their ability to generate ROS [29]. These properties are the reasons why AIEgens are widely used in biomedical fields such as biomolecular labeling, organelle imaging, cell tracking, antibacterial agents and disease diagnosis [30–46], and even make them excellent candidates to be PSs for PDT [47–50]. AIEgens-based PSs were reported by Liu and Tang et al. in 2014, which have attracted extensive attention [51]. Almost the same time, Hu et al. developed a new red-emissive bioprobe, TPE-red-2AP2H, which was successfully used to track the intracellular motion of the lysosomal protein transmembrane 4 beta (LAPTM4B) protein. Even better, ^1^O_2_ was generated under visible light, making this bioprobe potential for targeted photodynamic therapy as well [52]. In addition, novel therapeutic strategies that combine PDT with chemotherapy, photothermal therapy (PTT), immunotherapy or radiotherapy have been explored by researchers, so as to improve the efficacy of cancer treatment [53–59].

This review focuses on the recent studies of AIE-PSs in the treatment of various cancers. First, the principles and mechanisms of ROS generation are introduced, followed by the design and efficient manufacture of AIE-PSs. AIE-PSs-based PDT has been explored in preclinical trials for the treatment of tumors. Therefore, we summarize the potential of these AIE-PSs in cervical cancer, ovarian cancer, brain cancer, breast cancer, skin cancer, lung cancer, colorectal cancer, bladder cancer, colorectal cancer, bladder cancer and liver cancer from a clinical perspective. Meanwhile, we hope to promote the development of new anticancer strategies by exploring efficient PSs, including two-photon AIE-PSs and nano-functionalized AIE-PSs, to enable the application of AIE-PSs in clinical cancer therapy.

## Design of AIE-PSs for PDT

**Fig. 1:**
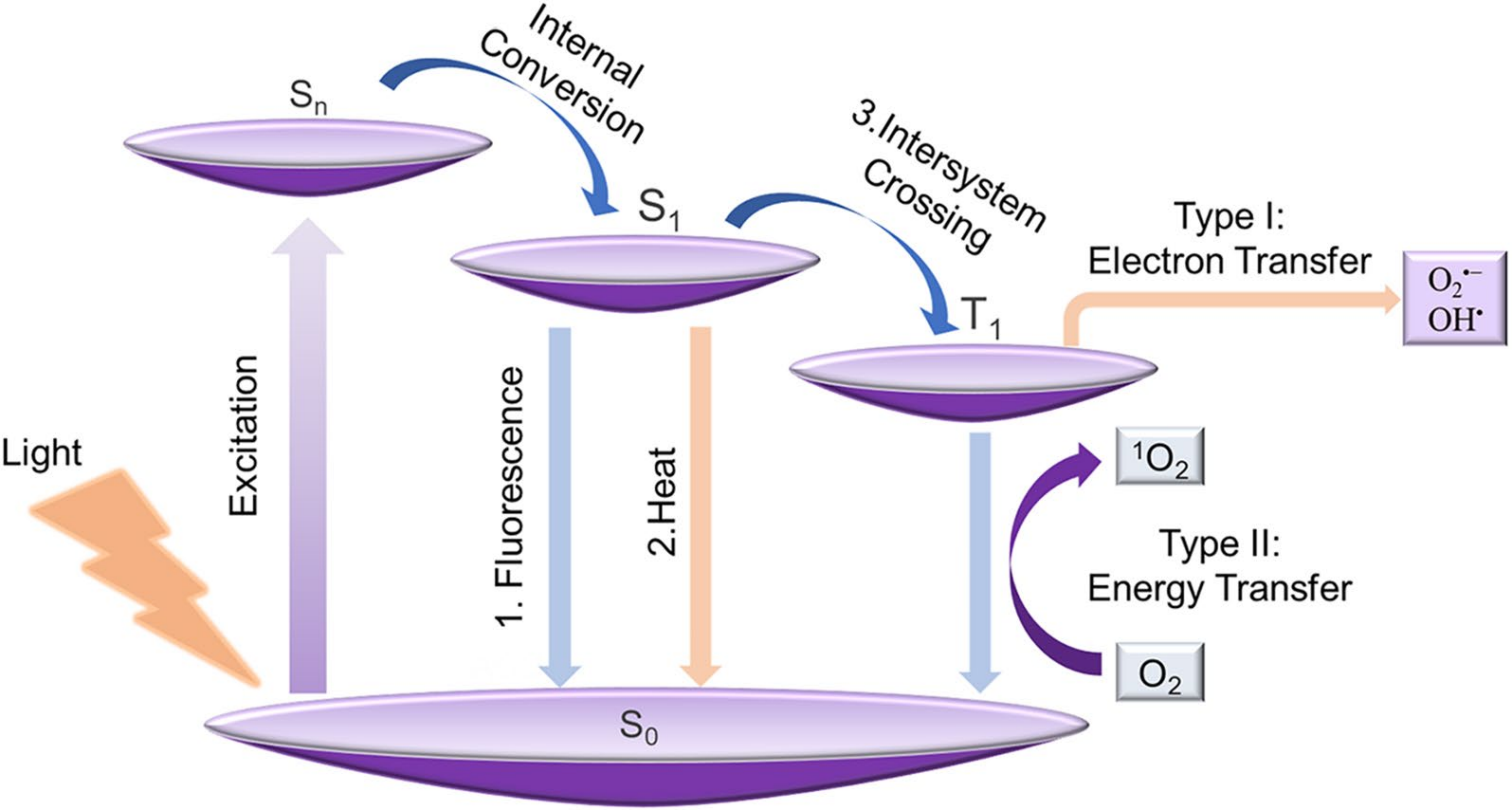
A Jablonski diagram explaining the working mechanism of type I and type II PSs

PDT is driven by physical processes of light that occurs between the excited photosensitizer and the surrounding oxygen or biological substrate environment [60, 61]. Efficient ROS generation is a decisive factor in the effectiveness of PDT, so improving the efficiency of ROS generation is one of the key factors in the design of AIE-PSs. Through the Jablonski diagram, we can clearly explain the mechanism of molecular fluorescence emission and production of ROS, which provides corresponding theoretical support for the rational design of PSs [62, 63]. As shown in Fig. 1, once molecules are excited by photons from the ground state (S_0_) to higher-energy orbitals (S_*n*_), the excited molecules will release energy during the internal conversion (IC), returning to the lowest singlet excited state (S_1_) [64]. There are three main pathways for S_1_ excitation, among which fluorescence is generated when molecules return from S_1_ to S_0_ via radiative decay, which can be used in the field of fluorescence imaging. In addition to fluorescence emission, non-radiative decay back to S_0_ also produces heat available for PTT. The final pathway is the transfer of S_1_ to T_1_ through intersystem crossover (ISC), in which electrons are transferred to the substrate to form toxic O_2_^•−^, OH• and H_2_O_2_ for type I PDT, or transfer their energy to the surrounding oxygen to form ^1^O_2_ at T_1_ state for type II PDT. Therefore, for the PS molecules designed for PDT, the efficiency of ISC in reducing the energy gap between the excited S_1_ and T_1_ states (ΔE_ST_) is the most critical factor.

**Fig. 2:**
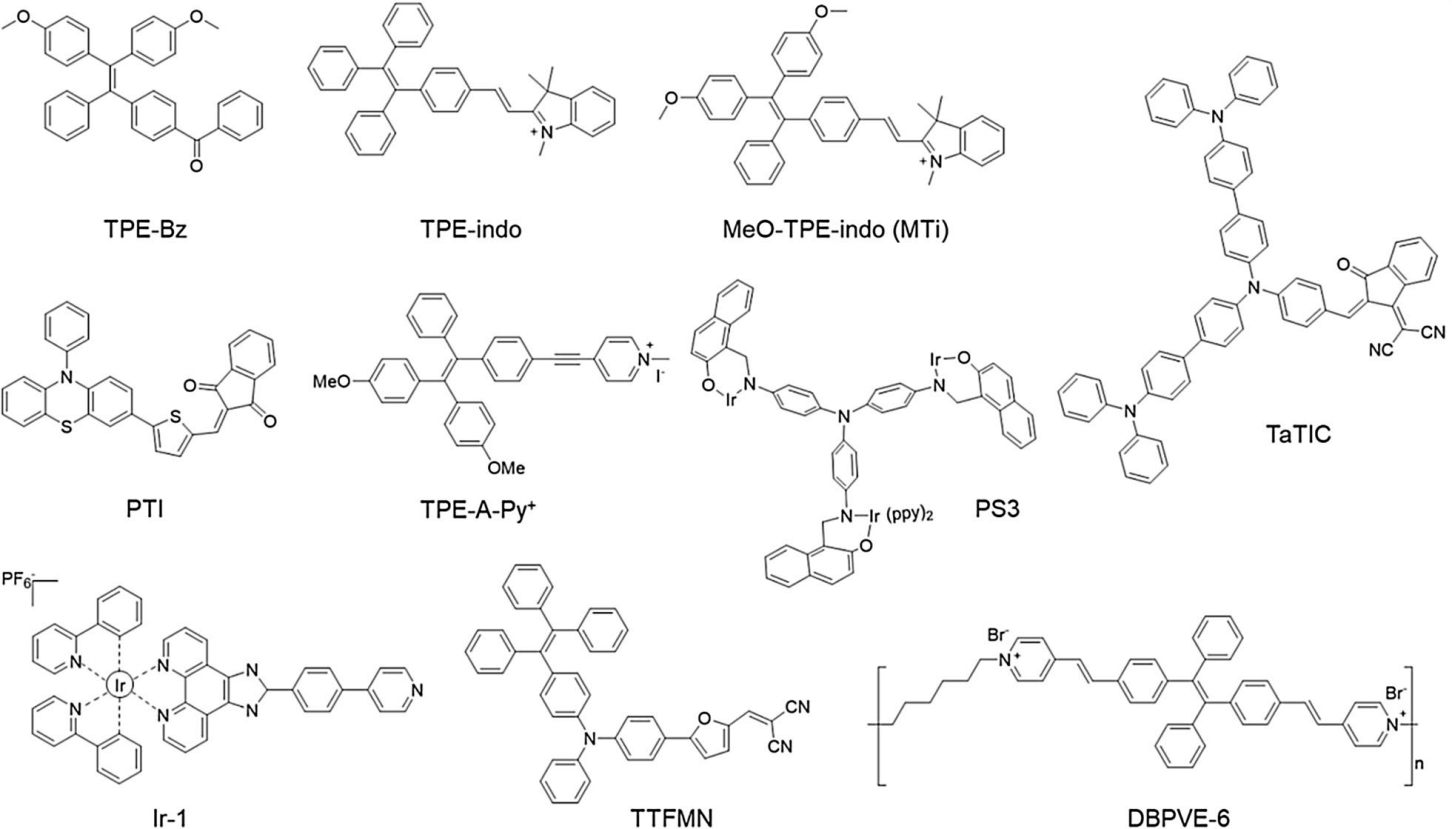
The molecular structures of AIE-PSs summarized in this review

The AIE phenomenon is mainly based on the mechanism of restricted intermolecular motion (RIM) [65–67]. When the molecules are in the aggregated state, the IC is prevented by restricted intramolecular motions, resulting in excited molecules to T_1_ through the radiative transition or ISC. PSs in the T_1_ state can undergo a series of reactions with surrounding substrates, thereby promoting the production of various ROS. The AIE-PSs generally enjoy promoted radiative pathways with suppressed non-radiative decay pathway in aggregation, leading to the production of more ROS, which has the potential to become the dominant molecule in PDT. Figure 2 summarizes the molecular structures of AIE PSs involved in this section.

### Manipulation of electron donor (D) and acceptor (A)

According to the above theoretical basis, we can know that ISC can be facilitated by reducing the ΔE_ST_ of the PSs to increase the generation of ROS [68]. Therefore, minimizing ΔE_ST_ is one of the key factors to achieve high ROS production efficiency. The most commonly used molecular design strategy is to manipulate the connection between the D and A to reduce the overlap between the distributions of the highest occupied molecular orbital (HOMO) and the lowest unoccupied molecular orbital (LUMO). Wang’s group designed a series of AIEgens based on tetraphenylethylene (TPE) skeletons to reduce ΔE_ST_, and the results of theoretical calculations and experiments are sufficient to prove the feasibility of this strategy [69]. They introduced methoxyl and positively charged indole groups on a TPE skeleton, with the methoxyl as D and positively charged indole acts as A group, to obtain the lowest ΔE_ST_ value. Compared to other molecules, the ΔE_ST_ of MTi is 0.633 eV, which is the lowest value among all molecules. This is an advantage of the molecule based on the D-A structure of methoxy and indole groups. Under the same white light irradiation (420–780 nm, 100 mW cm^−2^, 3 min), the rate of ^1^O_2_ generation was MTi > TPE-indo > TPE-BZ. The results indicated that the ^1^O_2_ generation among these AIEgens was consistent with the change trend of ΔE_ST_ values. To obtain AIE-PSs with high ^1^O_2_ efficiency, strong absorption and long wavelength emission, Lu et al. proposed a new molecular design strategy. The molecular design was started from TPT to achieve HOMO–LUMO separation for ^1^O_2_ generation. Subsequently, a new AIE-PS named TBT was designed using different A, 2,1,3-benzothiadiazole (BT) and 2-dicyanomethylene-3-cyano-4,5,5-trimethyl-2,5-dihydrofuran (TCF), linked together by a double bond as the combined A and further linked to the methoxy substituted TPE D. As the combined A of TBT shows a stronger electron withdrawing capacity than the single A, the charge transfer in TBT is stronger than that in TPT, achieving smaller ΔE_ST_ value and the goal of high efficiency of ^1^O_2_ generation [50]. So far, most of the reported AIE-PSs have been designed on the basis of this strategy, showing excellent ROS performances [70–76].

In addition, Tang et al. added different amounts of carbonyl groups (C = O) and cyanide groups (–CN) to the PSs design, in which the cyanide group is a strong electron acceptor, and the carbonyl group has a high spin–orbit coupling (SOC) effect due to its hybrid singlet and triplet transition electron configurations including n and π orbitals, thus resulting in decreased ΔE_ST_ value and increased SOC interaction [77]. Finally, these groups are bonded to the triphenylamine (TPA) electron donor via a small π bridge to obtain a series of triphenylamine-indole derivatives. To further enhance the electron D-A strength of the molecule, TPA was further modified by a series of electron donors to prepare a new molecule named TaTIC. The results confirmed that TaTIC achieved the highest ROS production ability compared with other synthesized molecules, even 2.42 times that of Rose Bengal (RB, a commercial PS). Therefore, their findings provided a new guidance for designing D-A type PSs to enhance photosensitization ability.

### Introduction of heavy atom or metal complex

In the past few decades, researchers have extensively exploited the indraught of heavy atoms (such as iodine and bromine) into the molecular structures to develop AIE-PSs with high-efficiency ROS. This is because heavy atoms can promote ISC process between the S_1_ and T_1_ states. Currently, three methods are existed for introducing heavy atoms, one of which is to introduce halogen atom via covalent bond. For example, Tang's group synthesized an AIE-active model PS (PI) with good ROS production efficiency and NIR emission capability [78]. To enhance the ISC process, a thiophene ring was inlet into the backbone of PI by the heavy atom effect to obtain PTI. The H2DCFH-DA probe is commonly utilized to assess the efficiency of ROS generation. Compared to PI, the fluorescence intensity of H2DCFH-DA rose rapidly to nearly 80-fold that of PTI (30-fold). This confirmed that the indraught of heavy atoms can indeed improve the production efficiency of ROS. The remaining two methods are the formation of the ionic bonds (e.g., TPE-A-Py^+^ [79]) and conjugated metal complexes with different ligands (e.g., PS3 [80], Ir-1 [81]). Unfortunately, the indraught of heavy atoms can lead to high dark toxicity, limiting their widespread applications.

### Design of type I AIE-PSs

**Fig. 3:**
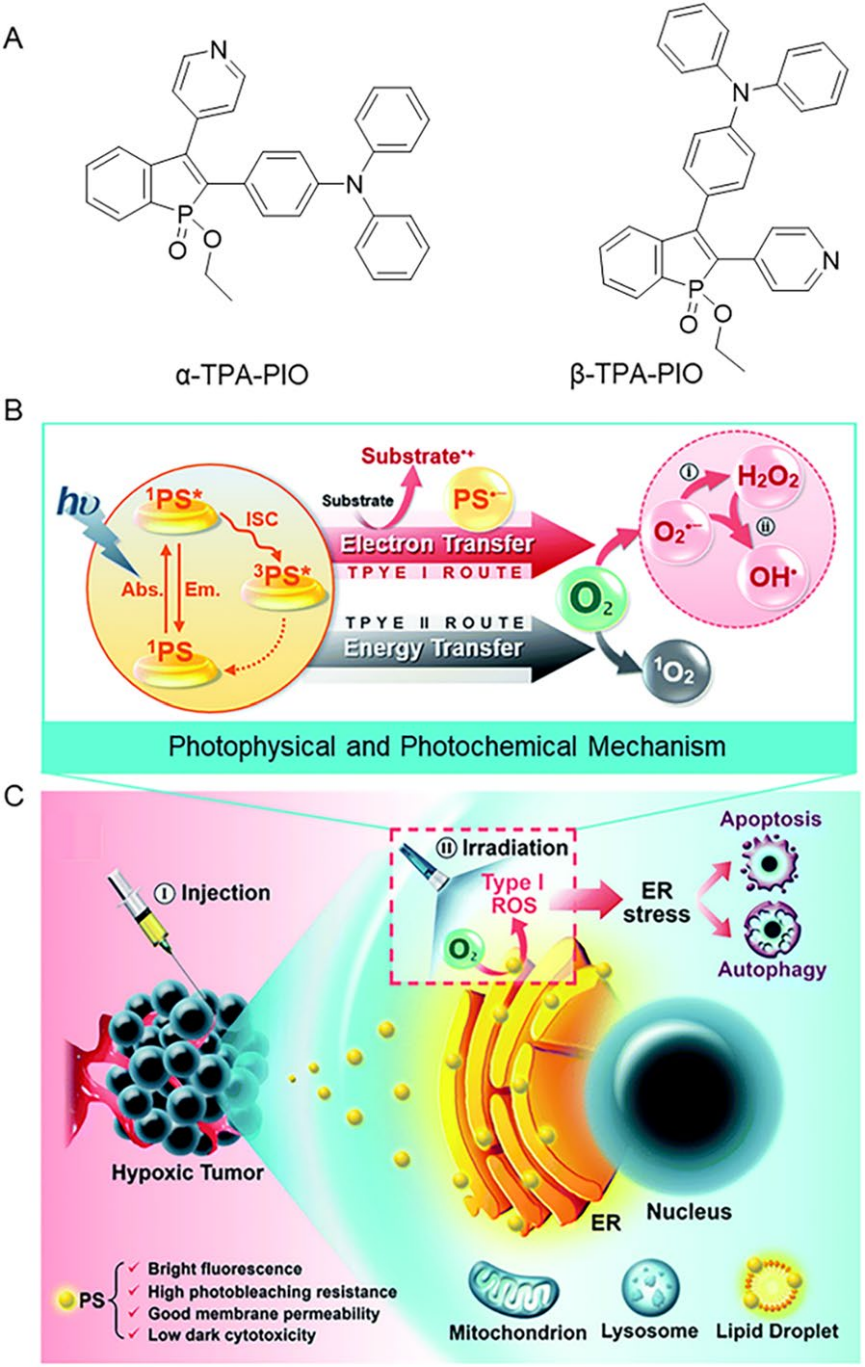
**A** The construction of α-TPA-PIO and β-TPA-PIO. **B** The schematic diagram of the photophysical and photochemical mechanisms. **C** The cytological process of PDT therapy mediated via PIO-based fluorescein. Reprinted with permission [84]. Copyright 2020, Royal Society of Chemistry

The majority of organic PSs have been reported to induce ROS via the Type II pathway, however, the production of ^1^O_2_ through the Type II pathway is highly relying on the presence of oxygen, which limits its effectiveness in severely hypoxic solid tumors [82]. The Type I process mainly generates low O_2_-dependent radical species, such as O_2_^•−^ and OH^•^, therefore, the design of Type I PSs becomes a key requirement for PDT. To address this challenge, Kang et al. designed the compound TTFMN with typical D-A structure [83]. Remarkably, the extreme electronic defects of the two cyano units on TTFMN are expected to favor the generation of Type-I ROS. Different ROS indexes were used to further determine the types of ROS generated by TTFMN. It was found that TTFMN showed the strongest signal strength for hydroxyphenyl fluoresce (the •OH generation indicator), while ABDA or SOSG (^1^O_2_ indicator) had almost no apparent signal change, manifesting its best generation ability of •OH. In addition, as shown in Fig. 3, Zhuang et al. reported another approach for Type-I PSs, obtaining phosphine oxide-based isomers α-TPA-PIO and β-TPA-PIO with efficient generation capacities of Type I ROS [84]. Not only that, they further collaborated to develop a series of tailored PSs with strong electron-withdrawing ability based on 9,10-phenanthrenequinone (PQ) [85]. The highly efficient generation of Type I ROS and excellent photothermal conversion ability are resourcefully integrated into these PQ-cored PSs to achieve Type I photodynamic and photothermal combined antitumor treatment. Other detailed studies on the photophysical and photochemical mechanisms of AIE-PSs provided a reference for the molecular design of AIE-PSs basing on the Type I mechanism [86–89].

### Polymerization-enhanced photosensitization

**Fig. 4:**
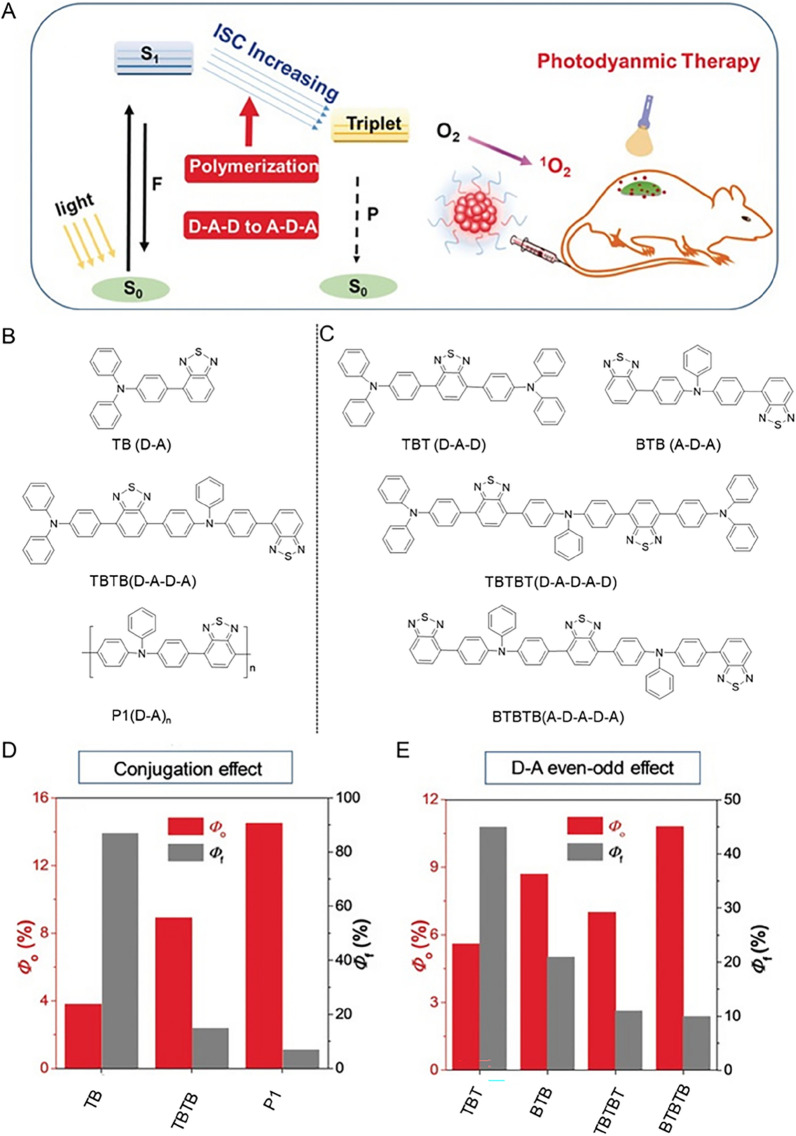
**A** Enhanced photosensitization for application in PDT. **B** Chemical structures of TB, TBTB and P1. **C** Chemical structures of TBT, BTB, TBTBT and BTBTB. **D** and **E** ^1^O_2_ quantum yield (*Φ*_*o*_) and fluorescence quantum yield (*Φ*_*F*_) of materials. Reproduced with permission [93]. Copyright 2018, Wiley–VCH

For the past few years, AIE molecules have also been introduced into polymers. Especially compared with PSs based on organic small molecules, AIE polymers possess prominent advantages such as signal amplification effect, multiple functionalization, as well as good processability, etc. [25, 90, 91]. The general strategy for preparing AIE polymers is to add AIEgens into the framework or as side chains of the polymer by modification and polymerization [92]. Excitingly, Liu et al. confirmed that conjugated polymers exhibited higher generation efficiency of ^1^O_2_ than their small-molecular counterparts (Fig. 4) [93]. By improving the strength of the conjugation, Zhu et al. synthesized four polyelectrolyte PSs with AIE fluorescence units, cationic units, conjugated units and aliphatic chains [94]. The ROS yields of DBPVE-6 under white light irradiation (400–700 nm, 60mW cm^−2^) were the highest, which may be due to its long aliphatic chain. This inspired us to design PSs by changing the conjugation strength and aliphatic chain length to achieve regulation of photocatalytic efficiency.

### Nano-engineering approaches

**Fig. 5:**
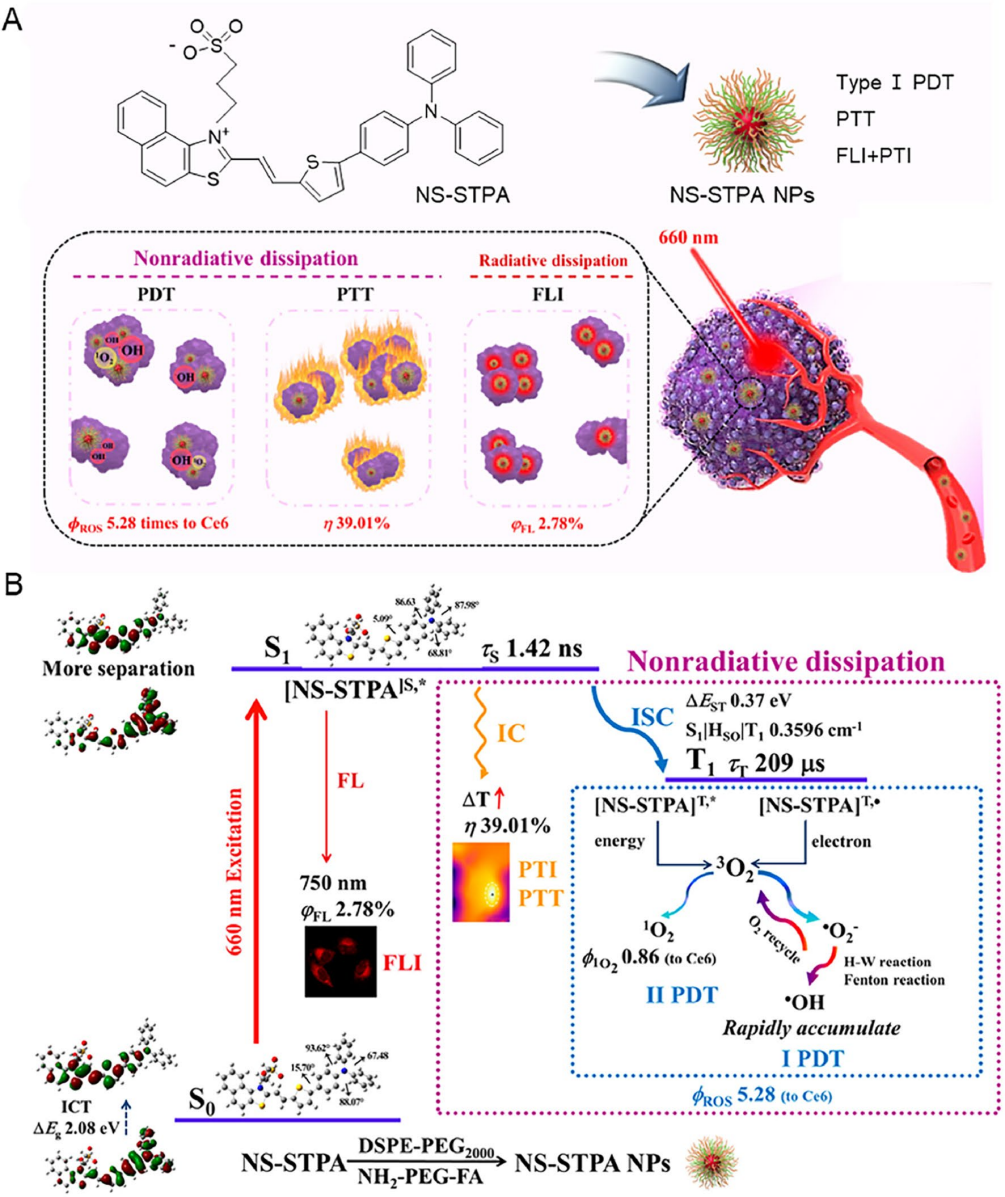
**A** The construction of NS-STPA and scheme of the application of NS-STPA NPs in fluorescence imaging-guided multimodal phototherapy. **B** The excited-state dissipation mechanism of NS-STPA NPs illustrated by the simplified Jablonski diagram. Reproduced with permission [95]. Copyright 2022, American Chemical Society

High ROS production could be guaranteed by molecular design to facilitate the ISC processes, but high ISC efficiency does not guarantee high ROS production in practical applications. Various inevitable problems will occur, such as poor solubility or destruction by polar solvents, when PSs are dispersed in the aquatic biological buffer. The preparation of nanoparticles is not only a simple method to overcome the hydrophobicity and biocompatibility problems of PSs, but also one of the strategies to improve ROS production. The long-life triplet states of nanoaggregates help to utilize the energy dissipation from S_1_ to T_1_ state, which is consistent with the principle of aggregation-induced ISC (AI-ISC). As shown in Fig. 5, Liu et al. synthesized NS-STPA by rigid coplanar grafting of flexible rotors and prepared them into nanoparticles (NS-STPA NPs), demonstrating that this method is an engineering approach to further improve ROS generation [95]. The apparent lifetime τ_T_ of triplet NS-STPA was 57.97 μs in DMSO, while its τ_T_ in nanoparticles was extended to 209.4 μs. Meanwhile, the ROS generation of NS-STPA and NS-STPA NPs was compared under the same conditions. The results indicated that the ROS generation property of NS-STPA NPs was significantly better than that of NS-STPA within a short irradiation time. These results demonstrated that the nanoengineered pathways had the ability to further enhance ROS production, which provided conditions for the clinical application of PSs. More encouragingly, the coated NPs can be modified on their surface by functional groups such as peptides, folic acid (FA), antibodies and membranes to obtain the desired bioactive functions [96–103], which would better satisfy the different clinical needs.

In addition, in order to improve the performance of PSs and reveal the possibility of its clinical application, the multi-functional modified AIE-PSs had also been extensively studied. The hydrophobicity of most AIEgens hinders their application in organisms, and using of polymer encapsulation is one of the strategies to overcome this problem. Polyethylene glycol (PEG) is a non-toxic polymer with good biocompatibility and excellent drug encapsulation efficiency [104, 105]. It avoids the removal of the reticuloendothelial system during in vivo circulation, resulting in reduced immunogenicity, which makes PEG the most commonly used polymer material for the preparation of nanoparticles (NPs). Meanwhile, Pluronic F127 is a highly biocompatible amphiphilic block copolymer that is commonly used as a coating for the preparation of nanoparticles [106]. To achieve the purpose of local aggregation of AIE-PSs in tumor, both targeted peptides (*e.g.* RGD peptide [107, 108]) and penetrating peptides (*e.g.* TAT peptide [109, 110]) contribute their own advantages to this end.

## AIE-PSs for different cancers treatment

### Ovarian cancer

Ovarian cancer has the highest mortality rate among gynecological malignancies, and the current treatment is surgery combined with chemotherapy regimen of paclitaxel/carboplatin [111]. At the beginning of treatment, most patients can effectively relieve, but the five-year survival rate is still less than 40% [112]. The main reasons are the high metastasis and high recurrence rates after treatment [113]. PDT effectively prolongs the survival of patients who cannot receive surgery, which has caused great concern. Tayyaba Hasan et al. have developed a photo-immune-regulating-liposome (PICAL), using an FDA-approved photosensitizer called Benzoporphyrin acid A (BPD) and an Cetuximab antibody for epidermal growth factor receptor (EGFR) to from Preformed Plain Liposome (PPL) [114]. The results showed that PPL increased the mortality of ovarian cancer cells. However, because of its low tissue penetration, these drugs can improve skin photosensitivity, reduce tumor specificity, lead to low human drug-resistant dose and tumor killing, so new photosensitizer is urgently needed to solve these problems.

**Fig. 6:**
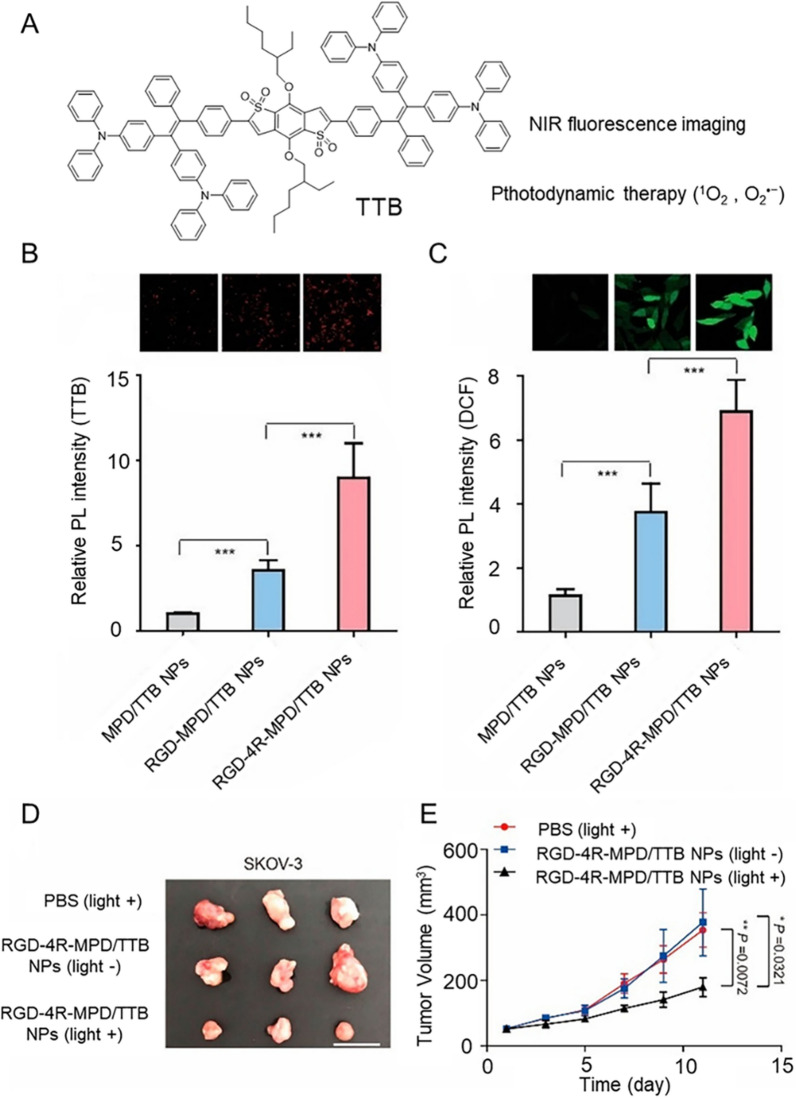
**A** Structure of TTB. **B** The relative PL intensity of SKOV-3 cells incubated with NPs. **C** Detection of intracellular ROS generation by DCFH-DA in SKOV-3 cells. **D** Tumor images, **E** tumor volume changes of SKOV-3 tumor-bearing mice after photodynamic therapy. Data were reported as mean ± SD and analyzed by two-sided Student’s t-test. *P < 0.05, **P < 0.01, ***P < 0.001, *n.s.* = not significant. Reproduced with permission [115]. Copyright 2020, American Chemical Society

AIE molecules are used for photodynamic treatment and image guidance, and have the advantages of avoiding the weak fluorescent signal caused by the ACQ effect. In order to further reduce background fluorescence interference and strong tissue penetration, an fluorogen including an electron-accepting benzo[1,2-b:4,5-b′]dithiophene 1,1,5,5-tetraoxide core and electron-donating 4,4′-(2,2-diphenylethene-1,1-diyl)bis(N,N-diphenylaniline) groups are used for PDT therapy and imaging, named TTB. Meanwhile, integrin α_ν_β_3_ has been reported to be an extremely important biomarker overexpressed in primary ovarian cancer cells, which has become a basis for the preparation of materials targeting ovarian cancer. Based on this, Dai and Li et al. simply encapsulated TTB into a biocompatible and water-soluble polymer matrix, 1,2-distearoyl-sn-glycero-3-phosphoethanolamine-N-[maleimide(polyethylene glycol)-2000] (MPD), in order to obtain good water-dispersible NPs [115]. To enhance the targeting ability of NPs to ovarian cancer cells, Mal- modified MPD/TTB NPs were prepared. Conjugation of MPD/TTB NPs to RGD peptide via the click reaction of SH and Mal groups resulted in RGD-MPD/TTB NPs that targeted the overexpressed integrin α_ν_β_3_ in ovarian cancer cells. Furthermore, an RGD-based modular peptide Arg-Gly-Asp-Phe-Gly-Gly-Arg-Arg-Arg-Arg-Cys (RGD-4R) carrying four arginine (R) was further designed. The RGD-4R peptide was grafted to MPD/TTB NPs through click reaction, which enhanced the ability of NPs to penetrate the cell membrane and facilitated the rapid entry of NPs into cells. The DCF signals were sequentially increased in SKOV-3 cells treated with MPD/TTB NPs, RGD-MPD/TTB NPs and RGD-4R-MPD/TTB NPs, while there was no significant difference in MCF7 cells (low expression of integrin α_ν_β_3_), suggesting that the ROS production efficiency of RGD-4R-MPD/TTB NPs was higher in SKOV-3 cells with overexpressed integrin expressing α_ν_β_3_ (Fig. 6). Finally, the ovarian cancer xenograft tumor model was established with SKOV-3 cells, and treatment was performed when the tumor volume reached 50 mm^3^. The tumors in the RGD-4R-MPD/TTB NPs (light +) groups were significantly inhibited, and H&E staining, TUNEL staining and IHC staining of the tumor tissues confirmed that the PDT based on RGD-4R-MPD/TTB NPs had good biocompatibility and could effectively suppress the growth of ovarian cancer. More noteworthy, RGD-4R-MPD/TTB NPs showed good therapeutic effects on integrin α_ν_β_3_-overexpressed cervical cancer (HeLa cells) and prostate cancer (PC3 cells). To reduce the recurrence in ovarian cancer patients, Dai designed a modular peptide probe (T_CD_TMP) comprising an AIE molecule (PyTPA) that can produce ROS efficiently [116]. It can self-assemble into NPs by loading miR-145-5p or VEGF-siRNA. This system reduces the recurrence rate of ovarian cancer in subcutaneous tumor models, abdominal metastasis models, and patient-derived tumor xenograft models, providing a new view for the surgical treatment of ovarian cancer.

To enhance tissue penetration, Wang et al. reported a two-photon cyclometalated iridium (III) complexes (Ir) fluorescent probe and further encapsulated into bio-compatible NPs with a penetration depth of 300 μm [81]. At the same time, it also possesses high photoinduced ROS generation efficiency, and mitochondrial targeting ability, which can interfere with redox homeostasis in vitro, leading to mitochondrial dysfunction and cell apoptosis. This is because intrinsic pathway apoptosis is the most common cell death discussed within the context of PDT, so the research on mitochondrial activity is particularly prominent [117]. The tumor volume of 100 mm^3^ of SKOV-3 tumor-bearing nude mice was treated with Ir-nanoparticles, and half of the nude mice tumors disappeared, whereas the tumor volume increased 26.2-folds in the PBS group. This provides a promising strategy for visualizing PDT therapy for ovarian cancer. The hypoxic environment at the tumor site greatly inhibits the therapeutic effect of PDT, so this article reports that the type I pathway PDT process. Not only that, the role of endoplasmic reticulum (ER) in PDT is not to be ignored. Immunogenic cell death (ICD) elicited by PDT is mediated through generation of ROS that induce ER stress [118]. The design of PSs for selective localization of the ER is the primary factor to determine the efficiency of PDT-induced ICD. Tang et al. constructed a PIO-based fluorophore that elicited ER stress and a dual cell death consisting of autophagy and apoptosis in vivo [84].

### Cervical cancer

Cervical cancer is the fourth commonest cancer in the world, which seriously affects women's life and health. Although it can be defended against with the HPV vaccine, persistent high-risk human papillomavirus (hrHPV) infection is the main cause of cervical cancer [119]. At present, the main treatment for cervical cancer includes radiotherapy, laser-based ablation, surgery and cold coagulation etc., but it still has many side effects [120]. PDT is extensively used in the treatment of malignant tumors due to its small trauma. A study on topical MAL-PDT reported that 75% (42/56) of cervical intraepithelial neoplasia (CIN) presented complete responses to MAL-PDT. For patients with CIN 2 and CIN 3, 90% are cured after two to three years of treatment [121]. Another article reported the topical administration of HAL suppositories in 47 patients with CIN 1 [122]. The results revealed that 57% of patients in the HAL-PDT group had resolution of CIN lesions at 6 months after PDT, compared with only 25% in the combined control group.

Several different combination therapies have shown excellent anti-cervical cancer efficacy, among which gene therapy (GT)/PDT is widely used. TTC-L-M-4/DOPE was prepared by Liu and Yang et al., and used to transfer siRNA (p53) with good results, indicating that gene silencing effect was better than Lipo2000 [123]. Meanwhile, TTC-L-M-4 had the ability to effectually produce ROS under light irradiation. Experiments in HeLa cell lines proved that p53 gene therapy combined with PDT greatly improved the efficacy of tumor therapy. Moreover, TTC-L-M-4/DOPE/p53 was injected into HeLa orthotopic tumor-bearing mice subcutaneously, and irradiated for 30 min, 24 h after injection, showing more effective tumor inhibition ability than other groups. Based on the same design principle, Tang et al. proposed a promising strategy for gene delivery in HEK293T cell lines [124]. Finally, combined with PDT therapy to achieve synergistic therapy, the killing effect on cancer cells was significantly enhanced. Tang et al. synthesized the PDT drug TBP-Au to avoid overexpression of the antioxidant system in cervical cancer [125]. Thioredoxin Reductase and its natural substrate, Thioredoxin, were one of the major antioxidant systems in cells, and inhibition of Thioredoxin Reductase will stimulate ROS production and induce apoptosis. The Au complex can effectively inhibit Thioredoxin Reductase, and the AIE-PS (TBP) can achieve imaging guidance and the specific treatment of ROS. The synergistic therapy of Au complex and TBP on Hela tumor-bearing nude mice under light displayed the ability to treat cervical cancer accurately.

Specific targeting of cervical cancer cells brings benefits for improving therapeutic efficiency. By recognizing the FA receptors (overexpressed in HeLa cells), Wen et al. synthesized FFM1 to target HeLa cells specifically, which showed outstanding capacities in target imaging and photodynamic killing of HeLa cells [126]. Targeting subcellular organelles can also effectively promote PDT. For example, Jiang et al. proposed a simple AIEgen, IQ-TPA, with mitochondrial targeting [127]. Preferably, it had bright two-photon fluorescence, enabling image-guided PDT with deeper penetration of tumors. All of this was demonstrated in HeLa cells. We know that the nucleus is identified as an ideal target for anti-tumor treatment because the DNA and some certain enzymes in the nucleus are the major causes of cancer and malignant proliferation. Wang and his collaborators developed MeTPAE, a nuclear targeting material [128]. MeTPAE can not only interact with histone deacetylases (HDACs) to suppress the proliferation of Hela cells, but also accurately destruct telomeres and nucleic acids via PDT, providing a new opportunity for the effective treatment of malignant tumors.

**Fig. 7:**
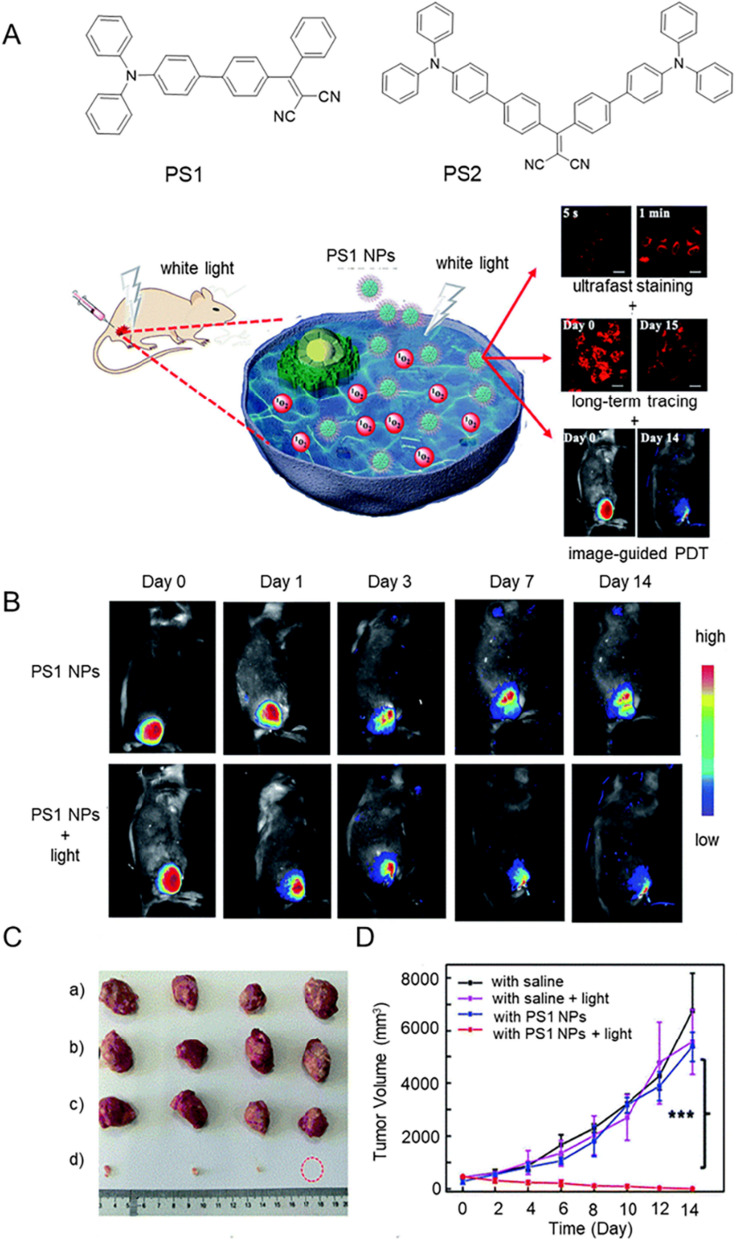
**A** Structural formulas of PS1 and PS2 and schematic illustration of PS1 NPs as PSs for “all-in-one” PDT. **B** Time-dependent fluorescence images of U14 tumor-bearing mice after intratumoral injection with PS1 NPs or with PS1 NPs + white light. **C** Tumors collected from different treatment groups. **D**Tumor volume changes by different treatment methods. (***P < 0.001, PDT *vs*. other groups). Reproduced with permission [129]. Copyright 2020, Royal Society of Chemistry

In order to effectively simplify cancer treatment, Zhang et al. developed multifunctional AIE-PSs [129]. As shown in Fig. 7, PS1 NPs were prepared by encapsulating PS1 into polymers, which exhibited bright red-emission, high quantum yield, good biocompatibility and suitable ^1^O_2_ generation ability. What's more, PS1 NPs incubated with HeLa cells with only gentle shaking for 5 s at room temperature to strongly illuminate the cytoplasm, showing ultra-fast staining and mild incubation conditions of PS1 NPs. Meanwhile, PS1 NPs could track cells for more than 14 days, demonstrating long-term tracking ability. Although the fluorescence intensity of U14 tumor-bearing mice decreased gradually, it still maintained at 42% of the initial intensity after 14 days. The relative tumor volume in the control group increased 10–13 times after 14 days, suggesting that radiotherapy alone or PS1 NPs had little effect on tumor growth. As expected, the tumor volume was significantly reduced in the PS1 NPs in white light irradiation group. This work resulted in an AIE-PS that combines image-guided PDT, ultra-fast staining, and long-term tracking capabilities, offering huge advantages for the clinical applications.

### Brain cancer

There are more than 120 types of brain tumors, and approximately 45% of primary brain tumors are gliomas, of which glioblastoma multiform (GBM) is the most common and aggressive tumor [130]. Regrettably, there are few effective treatments for these cancers, and the median survival rate for glioblastoma patients is only 14 months, even after aggressive surgery, chemotherapy and radiotherapy [131]. Chemotherapy is one of the most commonly used treatments for gliomas [132]. Unfortunately, the indiscriminate cytotoxicity of chemotherapy can lead to serious side effects as they fail to distinguish cancer cells from healthy cells, inhibiting fast-growing normal tissues and cells. Tumor cells are resistant to multiple other unexposed antitumor drugs with different structures and targets, which leads to multidrug resistance (MDR) in the treatment process. [133–135]. Such nonspecificity hinders the effect of chemotherapy and impairs the inhibition of tumor growth, metastasis, and recurrence [136–139]. With the innovation of treatment technology, it is possible to reduce the disadvantages of chemotherapy in cancer treatment, so that brain cancer can be better treated.

Appropriate brain imaging methods and corresponding excellent contrast agents are prerequisites for brain tumor treatment, which require good sensitivity and specificity, deep penetration, and high spatial and temporal resolution. Liu's group reported a novel NIR-II fluorescent molecule (TB1) with AIE property for in situ imaging of brain tumors [140]. TB1 was encapsulated in a polymer matrix and further modified with c-RGD to obtain targeted AIE dots, showing selective and specific tumor uptake, with a high signal/background ratio of 4.4 and a resolution up to 38 μm. More importantly, precise tumor-depth detection was achieved through non-invasive PA imaging of orthotopic brain tumors via the intact scalp and skull. To improve the resolution, Gao and Li collaborated to fabricate an albumin-based AIE nanoprobe, which has realized actively targeted NIR-II imaging of brain tumors and cerebrovascular imaging in a high-resolution (~ 70 μm) mouse model [141]. This provides a prerequisite for fluorescence imaging-guided surgery for cerebral ischemia and brain tumors, improving surgical efficacy and preventing recurrence and side effects.

**Fig. 8:**
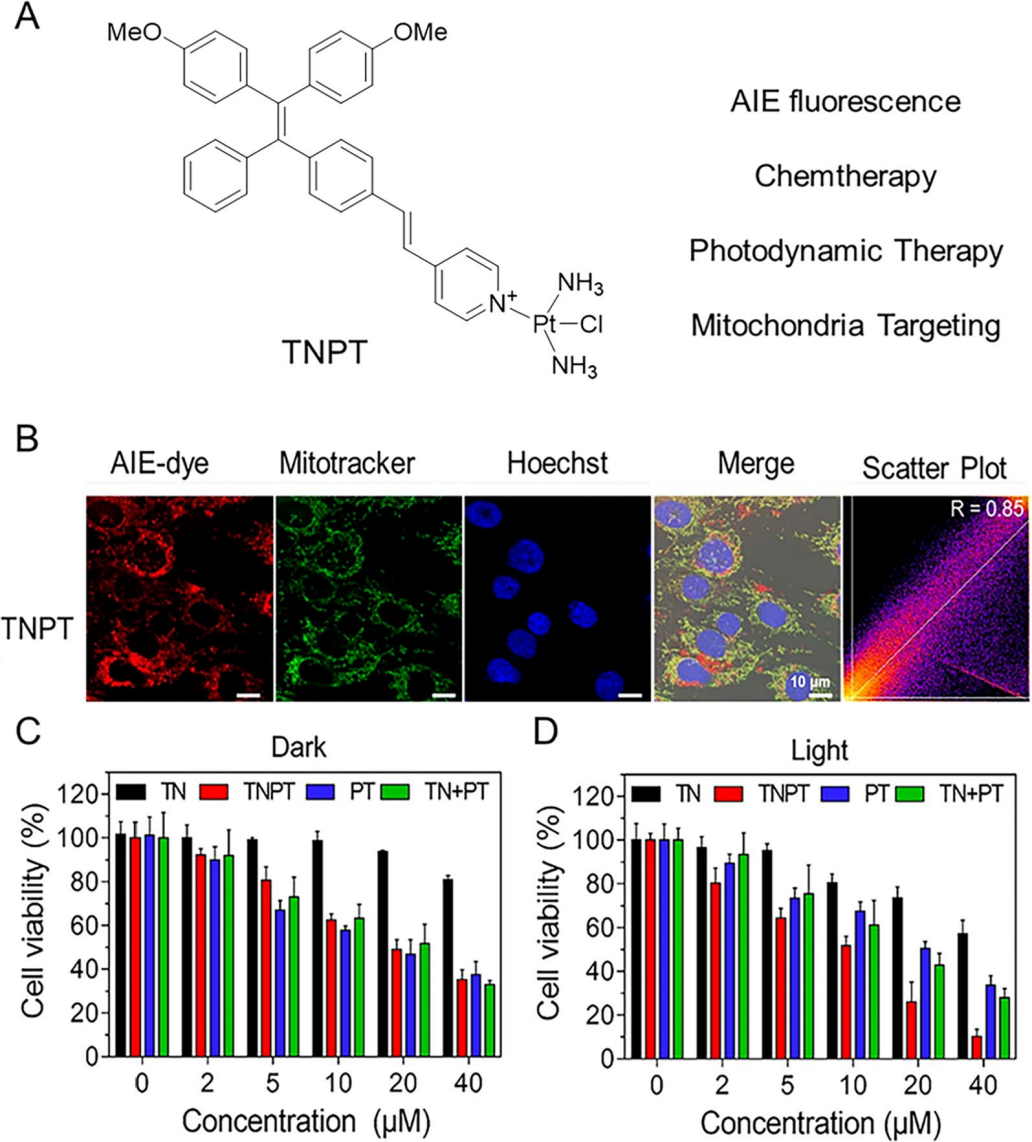
**A** Structure of TNPT. **B** CLSM images and overlapping coefficients shown in frequency scatterplots of C6 cells incubated with TNPT (5 μM) for 2 h. **C** Inhibitory effects of TN, TNPT, PT, and TN + PT on the C6 cell under dark and **D** light irradiation (15 J cm^−2^) at 50 mW cm^−2^ for 5 min. Reproduced with permission [145]. Copyright 2020, American Chemical Society

To circumvent the problems of chemotherapy, new therapies combining PDT and chemotherapy can efficiently eliminate extensive cancer cells and introduce minimal potential into MDR [142–144]. Guo and Wu skillfully combined AIE fluorophore and cisplatin to prepare mitochondria-targeted photosensitizer (TNPT) with AIE properties for collaborative anti-tumor therapy [145]. The chemotherapeutic efficacy of TNPT is similar to that of cisplatin, but its ability to generate ROS is higher than that of Ce6. In addition, TNPT has better biocompatibility than free cisplatin, significantly reduces cytotoxicity to normal cells, and shows selective uptake from cancerous cells rather than normal cells. Most importantly, TNPT has synergistic photodynamic and chemotherapy effects on C6 glioma cells, and was 2.4 times more effective than cisplatin under 15 J cm^−2^ white light irradiation (Fig. 8).

Another obstacle to glioma therapy is that the drug delivery system must cross different biological barriers during treatment, especially the blood–brain barrier (BBB) and blood-tumor barrier (BTB) [146]. Transferrin receptor (TfR) overexpression has been reported to exist in brain capillary endothelial cells and many malignant tumor cells [147]. In response, the T7 peptide was selected by Hu and Zhou et al. to design a delivery system across the blood–brain barrier because of its similar affinity with transferrin [148, 149]. An amphiphilic polymer (PLA-PEG-T7/TPE) with AIE properties was successfully constructed for drug delivery in tumor therapy. Finally, the drug TMZ was encapsulated into PLA-PEG-T7/TPE to construct nanoparticles (PLA-PEG-T7/TPE/TMZ). The inhibition of cell proliferation suggested that the drug micelles had dose-dependent cytotoxicity to LN229 cells. Meanwhile, the therapeutic effect of PLA-PEG-T7/TPE/TMZ was the best in the in vivo anti-tumor experiments.

Brain-targeting apolipoprotein E peptide (ApoE) is a targeted functional peptide that promotes effective BBB penetration [150]. Brain-targeted near-infrared IIb (NIR-IIb) AIE nanoparticles were developed by Wang et al., and ApoE was then grafted onto these nanoparticles, termed ApoE-Ph NPs [151]. A band-pass filter with a wavelength of 1550 nm could be utilized to monitor the in vivo biological distribution and accumulation of nanoparticles in an in-situ glioma model, overcoming previous limitations in wavelength range and equipment. Under 808 nm laser irradiation, ApoE-Ph NPs exhibited high PTT efficiency and significantly improved the survival rate of in-situ GBM mice. Fortunately, recent clinical studies have shown that immune cells can perform immune surveillance in the central nervous system to cross the blood–brain barrier. Even more impressively, Natural killer (NK) cells represent a variety of proteins for tumor recognition and has been considered for cancer immunotherapy. Deng and his collaborators developed a natural-killer-cell-mimic nanorobots (NK@AIEdots) by covering the AIE-active polymer endoskeleton PBPTV with NK cell membranes [152]. It is noteworthy that NK@AIEdots can pass through the BBB in a self-help manner and specifically accumulate in the complex brain stroma of brain tumors. This greatly improves the efficiency of in situ transcranial-scalp fluorescence imaging and PTT. It turns out that these NK@AIEdots did greatly inhibit tumor growth under NIR light. All of these strategies show great potential in active transmission of BBB, which lays a foundation for the treatment of brain cancer.

### Skin cancer

There are two types of skin cancer, including malignant melanoma (MM) and non-melanoma skin cancer (NMSC) [153, 154]. The incidence of both MM and NMSC has increased over the past few decades, and the incidence of all skin cancers is much higher than that of almost any other type of cancer. Cutaneous malignant melanoma (CMM) is the most common, fastest-growing malignancy in Caucasians, and the most aggressive form of all skin cancers diagnosed [155]. Chemotherapy used immediately after the surgery is an essential element and the key treatment modality in the available treatment options for skin cancer. Unfortunately, traditional chemoprevention regimens have major problems with lack of response to treatment and associated side effects [156]. Therefore, much more effective anti-cancer drugs and treatments are needed. Currently, immunotherapy and targeted therapy have been developed, and other treatments are being investigated [157].

**Fig. 9:**
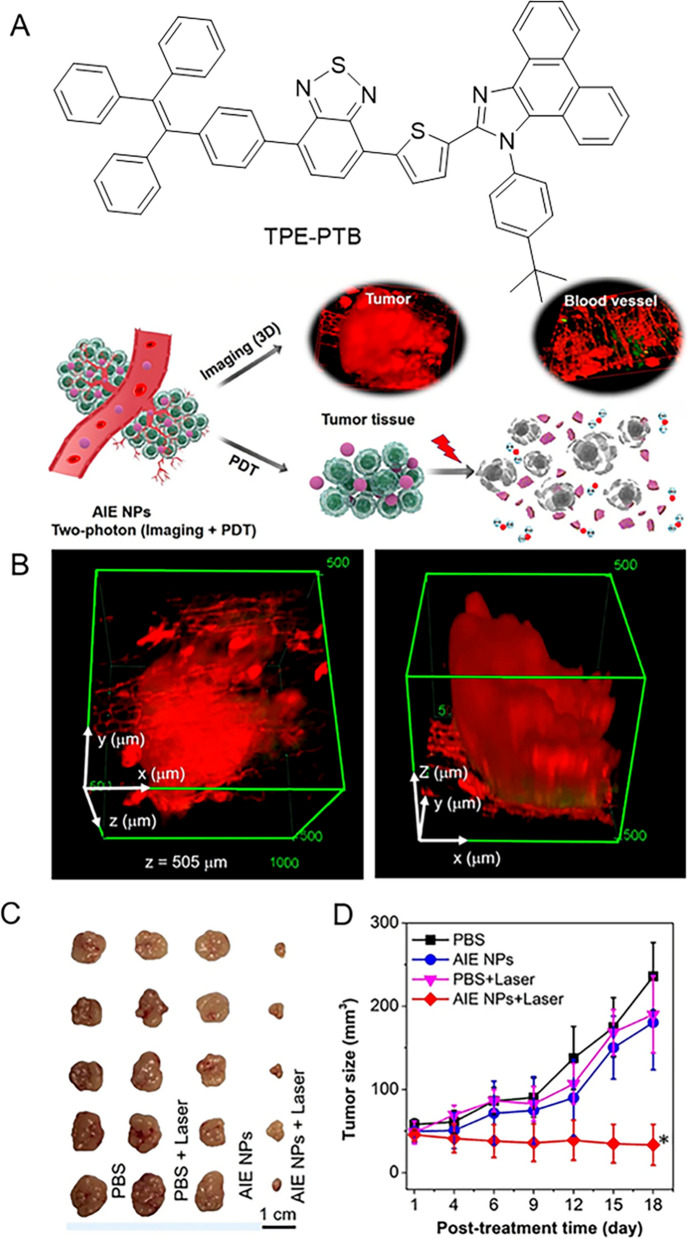
**A** Structure of TPE-PTB and AIE NPs for TPFI guided PDT application. **B** Two-photon 3D reconstructed images of the tumor tissue. **C** Photograph of the resected tumors in different treatment groups. **D** Tumor growth curve during the treatments. The data show significant statistical difference between the laser irradiation group after AIE NPs-treatment and the laser irradiation group after PBS treatment. (*P < 0.05). Reproduced with permission [160]. Copyright 2020, American Chemical Society

MM has a high tendency to metastasize, and once melanoma has spread beyond its original location, surgical treatment becomes extremely difficult, while resistance to conventional chemotherapy and radiation has increased dramatically [158]. Happily, PDT is emerging as an effective way with precise spatiotemporal control for cancer treatment and may be a promising treatment for melanoma. Tang’s research group developed an AIE molecule named DCQu, showing extremely high ^1^O_2_ generation efficiency and near-infrared two-photon activity [159]. DCQu not only selectively targets cancer cells without using any biomarkers or additional modifications to target ligands, but also specifically stains mitochondria. Under white light irradiation, DCQu showed significant dose-dependent toxicity, with cancer cell viability decreasing to 9% at 10 μM. The survival percentage of mice treated with “Ce6 + light” remained ~ 60% at day 45, while that of “DCQu + light” reached ~ 80%, indicating that the PDT application of DCQu prolonged the survival time of B16 melanoma-bearing mice. In order to more accurately locate melanoma in space, as shown in Fig. 9, Li and Tang jointly proposed that lipid-encapsulated AIE nanoparticles (AIE NPs) could be used as laser imaging agent for spatiotemporal imaging of tumor tissues with penetration depth up to 505 μm in A375 tumor-bearing mice models [160]. Not only that, AIE NPs can simultaneously generate ^1^O_2_ and •OH to induce tumor ablation. Finally, AIE NPs could be effectively eliminated from the mice after imaging and treatment.

The incidence of MM is relatively scarce compared to the highest mortality rate of all skin cancers. Among Caucasians, the incidence of NMSC is much higher than that of MM, so the treatment of NMSC also requires further studied [161]. NMSC includes basal cell carcinoma (BCC) and squamous cell carcinoma (SCC), and so on [162]. Wang and Zhu et al. collaborated to develop an AIE-based therapeutic agent (TPA3) that is phototoxic to cancer cells and A431 (human basal cell carcinoma) tumor-bearing nude mouse under irradiation of white light [163]. The photocytotoxicity of TPA3 in cancer cells was detected by MTT assay. Encouragingly, an extremely low concentrations (5 μM) of TPA3 resulted in the death of about 70% of cancer cells after LED irradiation for 10 min, while normal cells were not significantly injured. This fully demonstrated that TPA3 had the advantage of being specifically selective for cancer cells, prompting further exploration in vivo. TPA3 was injected into A431 tumor-bearing nude mouse model 24 h later. By comparing the fluorescence signals of tumor and normal organs, TPA3 was found to mainly concentrate in the tumor site. When the tumor volume reached ~ 50 mm^3^, the mice were randomly divided into four groups and given different treatment regimens. TPA3 (100 µL 200 mm^−3^) was injected into tumor-bearing mice after 30 min of irradiation with LED light (200 mW cm^−2^, 10 min). Additional illumination was given every 3 days, and the relative tumor volume was measured 14 days later. Compared with other groups (PBS, PBS with light, TPA3), the TPA3 with light group had a significant inhibitory effect on tumors, indicating that TPA3 had a good PDT treatment effect on tumors under LED light. More excitingly, in addition to its effective PDT, TPA3 provides in-situ monitoring of dynamical mitophagy process involving mitochondria, AVs, and lysosomes, which will effectively avoid overtreatment by providing a real-time self-monitoring system for assessing the efficacy of late cell apoptosis.

### Lung cancer

Lung cancer is a major cause of cancer-related morbidity and mortality and has been increasing over the past decades, becoming a great challenge for health management department and mankind [164]. Of concern was that lung cancer accounts for about 20% of all cancer diagnoses. Until now, treatment has generally been determined according to the stage of lung cancer. There is little dispute, for example, that surgery is the preferred treatment for stage I to II lung cancer for eligible patients [165]. Radiotherapy is mainly used for early and intermediate non-small cell lung cancer (NSCLC) [166]. Systemic therapy, including chemotherapy, targeted therapy, and immunotherapy, is the dominant treatment strategy for patients diagnosed with advanced/metastatic lung cancer [167]. In line with changes in medical practice related to cancer screening and/or treatment, advances in the treatment of lung cancer has intensified, however, more approaches to treatment must be needed.

The specific recognition of overexpressed antigens on the surface of lung cancer cells provides a great opportunity for accurate diagnosis and treatment of lung cancer. Cetuximab (C225) has been proved to be an effective therapeutic agent by specifically targeting EGFR factors overexpressed in lung cancer [168]. Based on the high specificity and affinity of C225, *t*-BuPITBT-TPE-C225 NPs were obtained by modifying *t*-BuPITBT-TPE encapsulated in biocompatible DSPE-PEG with C225 [169]. *t*-BuPITBT-TPE-C225 NPs can be used to target non-small cell lung cancer cells, which has been validated by confocal microscopy and flow cytometry experiments. Su et al. also utilized C225 coupled with TPENI NPs to achieve specific fluorescence imaging and detection of non-small lung cancer cells [170]. Low-density lipoprotein receptor (LDLR) that was typically upregulated in A549 cells (Human non-small cell lung cancer cells), was also a target for cancer cells. Based on this, Wang et al. designed and synthesized a novel AIE-PS (TPA-DPPY) that generates ROS under white light irradiation and specifically encapsulated it into low-density lipoprotein (LDL) particles as a PS for photodynamic killing of A549 cells [171]. According to quantitative analysis of cell viability data, TPA-DPPY exhibited obvious phototoxicity to A549 cancer cells, with killing efficiency up to about 88%. Interestingly, the ROS generation process is accompanied by significant changes in TPA-DPPY luminescence, such as enhanced luminescence and blue shift emission, allowing real-time fluorescence monitoring of the PDT process. This capability is extremely important because it provides immediate feedback on treatment outcomes and has great potential in a multifunctional PDT.

Metallic drugs have been used to treat cancer for more than 50 years, since the advent of cisplatin [172]. However, cancer cells can develop metal drug resistance after multiple rounds of treatment, greatly limiting the efficacy of treatment, which is no exception for lung cancer treatment [173]. To overcome cisplatin resistance, Su et al. proposed a new strategy to solve the problem through mitochondrial dysfunction according to the anticancer mechanism of cisplatin (cis-Pt) [174]. Two mitochondria-targeted AIE molecules, DP-PPh_3_ and TPE-PPh_3_, demonstrated superior ability to overcome cisplatin resistance in lung cancer cells (A549R) by altering drug metabolism (up-regulation of influx CTR1 and down-regulation of efflux MRP2) and blocking autophagy flux (failure of the degradation of autophagosomes). Meanwhile, DP-PPh_3_ and TPE-PPh_3,_ acting as PSs, could induce ROS production, resulting in the destruction of mitochondrial structure and impaired glycolytic metabolism. In addition, the anticancer effects of these two molecules were verified in 3D multicellular tumor spheroids (MCTSs) of A549R cells and tumor-bearing nude mice. This strategy not only promotes the PDT effect on cancer cells, but also provides a new pathway to drug resistance for metal drug therapy.

More photosensitizers are being developed to treat lung cancer, Deng et al. developed an AIE-PS BODIPY-TPA, which could be transformed into relevant nanoparticles (BODIPY-TPA NPs) through self-assembly process, and further studied the photodynamic toxicity of the particles to A549 cells [175]. It was confirmed that the IC50 of BODIPY-TPA NPs was 28.45 μg mL^−1^ under a single laser irradiation of 635 nm. Zheng and collaborators prepared an NPs-targeting mitochondria using a novel AIE cross-linked copolymer (PAIE-TPP), which was highly cytotoxic to A549 cancer cells with a survival rate of only 4% under an ultralow white light power intensity of 10 mW cm^−2^ [176]. These results indicate that particles coated with AIE-PSs can be used as a class of safe and multifunctional nano phototherapy drugs in the field of PDT for lung cancer cells, which has become the basis for further exploration in clinical experiments.

**Fig. 10:**
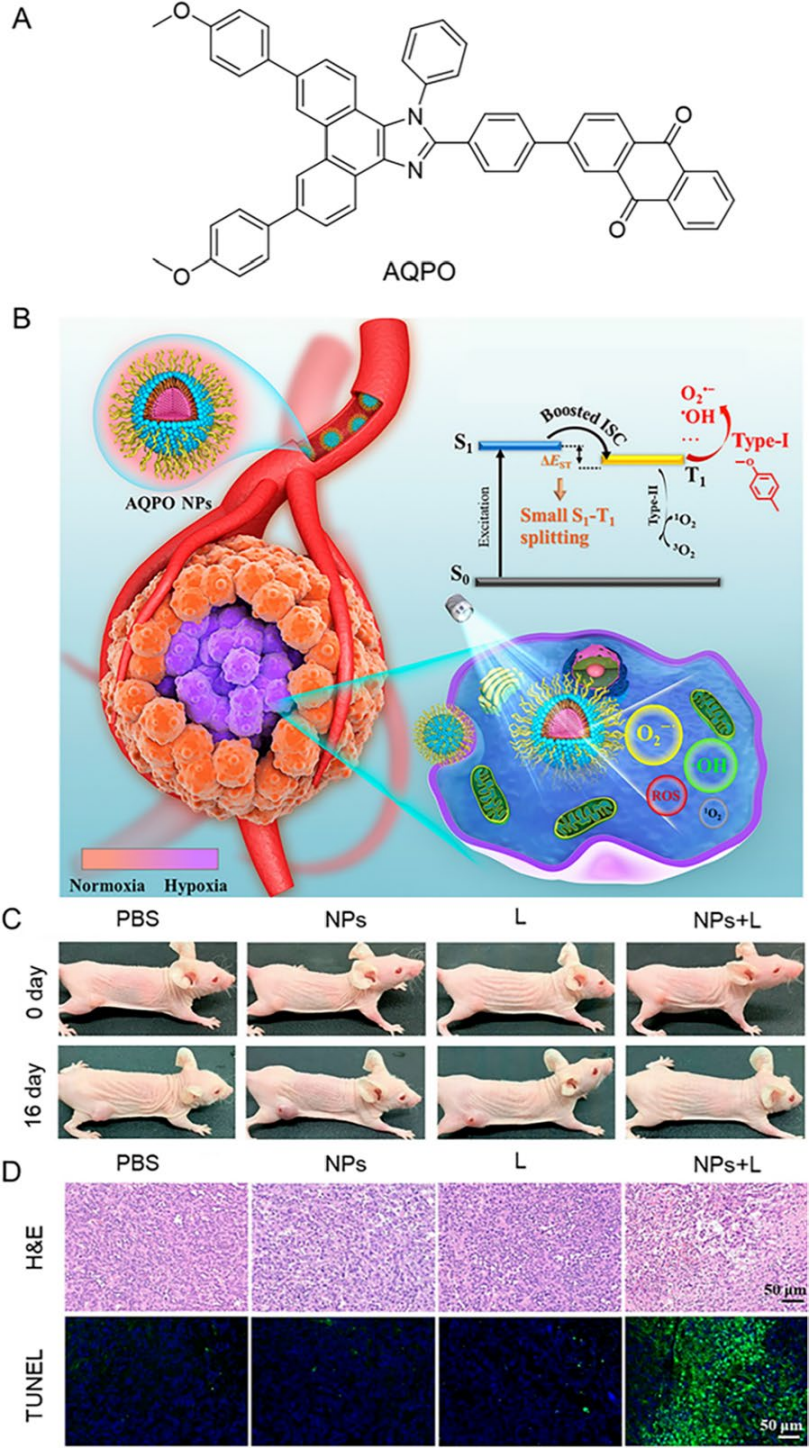
**A** Structure of AQPO. **B** Schematic diagram illustration of AQPO NPs for cancer treatment. **C** Pictures of A549 tumor-bearing mice in different treatment groups on the 0 day and 16th day, respectively. **D** H&E and TUNEL staining of tumor sections of mice in different treatment groups. Reproduced with permission [177]. Copyright 2022, American Chemical Society

Based on the great advances in AIE-PSs at the cellular level, the researchers continue to study its effects in A549 tumor-bearing mice. As shown in Fig. 10, Xiao et al. demonstrated the synthesis of a novel AIE (AQPO) and its assembly of nanoparticles (NPs) to promote the increase of O^2−^ and •OH generation efficiencies [177]. Using A549 tumor-bearing mice as models, they investigated the PDT effect of AQPO NPs in vivo. Mice were randomly divided into 4 groups: PBS, PBS + light (L), AQPO NPs (NPs) and AQPO NPs + light (NPs + L). After 16 days of different treatments, significant suppression of tumor growth was observed in the group of NPs + L, compared with PBS, L and NPs groups, which was mainly due to its superior PDT and hypoxia-overcoming effect of AQPO NPs. Meanwhile, H&E staining showed that the NPs + L group exhibited more apoptotic and necrotic cells compared with the other three groups. These results further indicated that AQPO NPs could effectively eliminate tumor cells under white light irradiation and had the excellent anti-hypoxia PDT effect. In addition, Cao et al. constructed a biodegradable polymer with AIE characteristics, which had mitochondrial targeting ability [178]. This polymer improved the efficiency of co-encapsulated photosensitizers and the therapeutic index of cancer cells in vitro and in vivo, contributing to the photodynamic therapy of lung cancer.

### Breast cancer

According to the latest data from the American Cancer Society, which estimated the numbers of cancer cases and deaths in the United States, the incidence of breast cancer in women continued to rise slowly (0.5% per year) between 2014 and 2018 [179]. Despite encouraging advances in breast cancer treatment and related 5-year survival outcomes, the treatment of metastatic breast cancer remains a major challenge [180]. For breast cancer diagnosed locally, which accounts for approximately 90% of all cases, the main treatment is surgery to achieve complete resection of the primary tumor [181]. However, even the most advanced surgeries and treatments can not prevent breast cancer recurrence and metastasis. Therefore, the first step in local treatment of cancer cells is surgical resection, usually followed by radiation therapy of the tumor bed [182]. Finally, Systemic drug therapy is administered with the aim of eliminating any residual cells to prevent metastatic recurrence. Although the current standard of treatment includes a combination of surgery, radiation and drugs, other treatments need to be introduced to treat breast cancer completely [183–185]. The excellent performance of PDT in clinical practice has undoubtedly become an interesting new research approach.

**Fig. 11:**
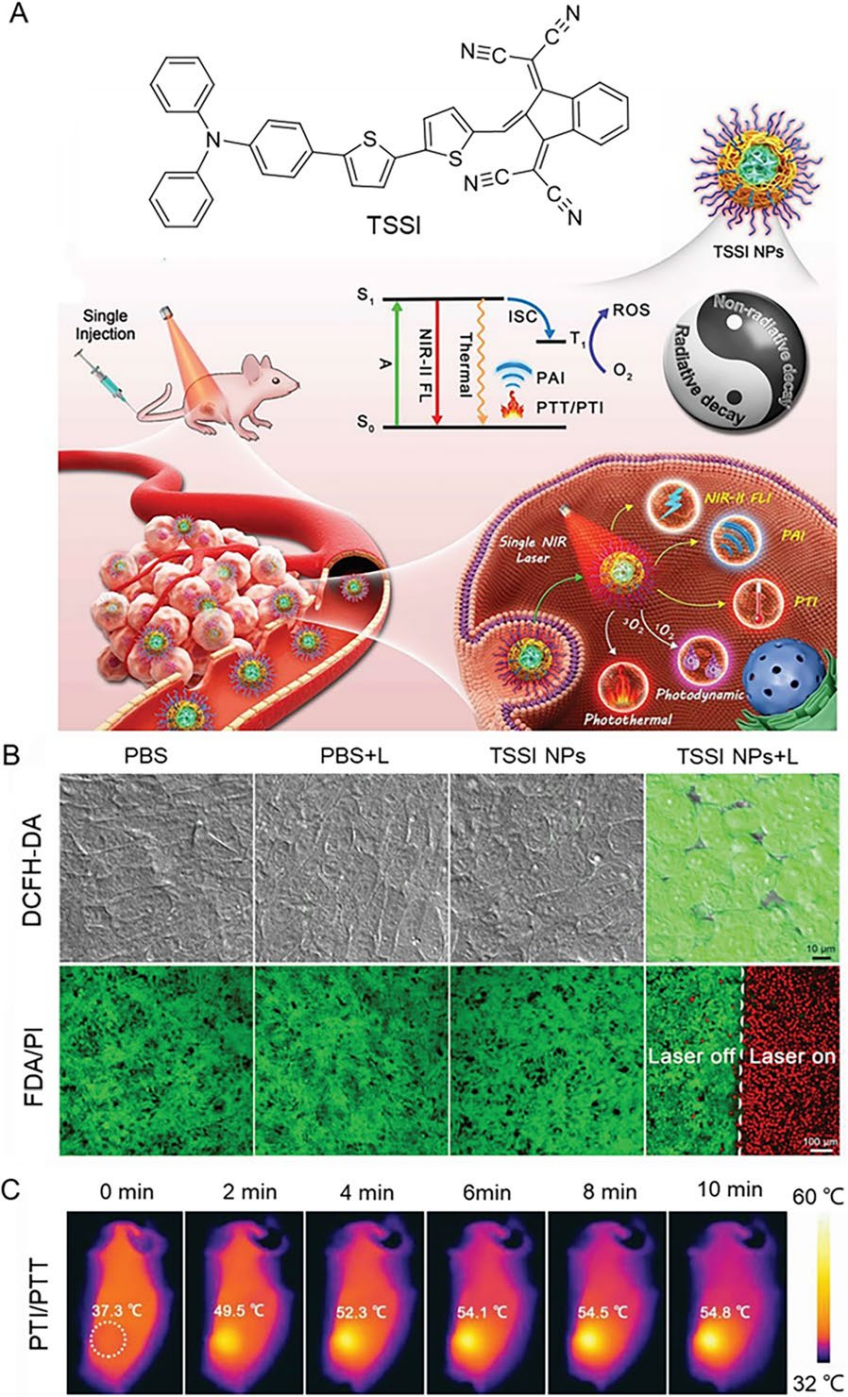
**A** The structure of TSSI and the process of TSSI NPs in the treatment of tumor with PDT-PTT synergistic therapy under the guidance of NIR-II FLI-PAI-PTI trimodal imaging. **B** Intracellular ROS level and live/dead cell staining of 4T1 cells after treatment under different conditions. **C** Thermal images, heating temperatures (at tumor sites) of 4T1-tumor-bearing BALB/c nude mice during continuous NIR irradiation at 12 h postinjection of TSSI NPs. Reproduced with permission [192]. Copyright 2020, Wiley–VCH

Currently, surgical resection is the most direct way to treat solid tumors, but small lesions remain after treatment. Therefore, combined with PDT after resection is a favorable choice to cure the disease. In collaboration with Dai et al., Jiang developed a single AIE molecule to achieve NIR fluorescence surgical navigation in breast cancer mice, and some microscopic residual tumors are further completely ablated by PDT and PTT to maximize the death of tumor cells and tissues. With this combination of image-guided surgery, PDT and PTT, survival rates of up to 90% can be achieved [186]. Besides, there is a great demand for therapeutic anticancer drugs with multiple therapeutic functions for breast cancer. Platinum (Pt)-based anticancer drugs are still commonly used chemotherapy drugs in the treatment of breast cancer. Non-toxic Pt(IV) prodrugs have been subsequently developed, and are widely used due to their reduced side effects. Su and collaborators developed a triphenyl phosphorus (TPP) modified AIE-based Pt(IV) prodrug ACPt, which was for the image-guided chemotherapy [187]. ACPt has high cytotoxicity to human breast cancer cells and can induce the increase of ROS. Specifically, the toxicity of ACPt to MCF-7 cells was about 2.5 times that of free AC ligand, and around 5.5 times to the CDDP, suggested that ACPt has a strong synergistic effect on MCF-7 cells. Furthermore, the combination of PDT and PTT showed promising advantages in the treatment of breast cancer. Two novel AIE-active fluorinated compounds, DPMD and TPMD, had been designed by Liu et al. [188]. Taking TPMD as an example, nanoparticles were constructed by encapsulating it into amphiphilic polymer F12, named as TPMD NPs. It is noteworthy that TPMD NPs had a great advantage in PDT due to their efficient production of ROS, while their excellent photothermal effects could be used for photothermal imaging, photoacoustic imaging, and PTT. The cooperation of PDT and PTT is considered as successful system in synergistic cancer therapy to achieve synergistic effects with boosted therapeutic outcomes [189, 190]. The main reason is that the PDT efficacy can be photothermally enhanced, wherein the PTT could improve the oxygen supply in the tumor tissue through raising the blood flow rate, which conversely further eliminates the heat-resistant tumor cells in PTT [191]. It was found that combining TPMD NPs injection with laser irradiation (650 nm NIR laser, 600 mW cm^−2^) at 24 h postinjection, the tumors of 4T1 tumor-bearing mice were completely eliminated at day 2 after treatment, without recurrence or regeneration. This fully indicated that AIE had high efficiency in the treatment of PDT and PTT combination therapy, and sufficient clinical application potential. Aiming at multifunctional therapy that achieves the comprehensive function of multi-diagnostic imaging and synergistic therapy, Zhang et al. ingeniously designed and constructed a simple and mighty “one-click” therapy based on an AIE-active PS (TSSI) [192]. It exhibited bright NIR-II fluorescence emission, high photothermal conversion efficiency and highly efficient ROS generation (Fig. 11). The unprecedented performance of PDT-PTT co-therapy guided by NIR-II fluorescence imaging (FLI)-photoacoustic imaging (PAI)-photothermal imaging (PTI) trimodal-imaging was confirmed by the diagnosis of 4T1 tumor and the outcomes of complete tumor elimination.

Human epidermal growth factor receptor 2 (HER2) is over-expressed in more than 20% of breast cancer cases. Therapies targeting HER2 have yielded unparalleled clinical benefits for a subset of breast cancer patients. Zhang et al. successfully applied supramolecular materials and transformable peptides to control HER2 signaling in breast cancer cells [193]. In vivo study of MCF-7/C6 and BT474 xenograft models in mice had shown that this therapy is effective in treating HER2 + breast cancer xenografts. In addition, nanoparticles formed by AIE-PSs contribute to PDT for breast cancer. Liu et al. encapsulated a single AIE-PS into F-127 to form nanoparticles (TBTDC NPs), which exhibited good biocompatibility, highly specific targeting of lysosomes, and impressive tissue penetration (up to 300 μm) [194]. Cell viability of MCF-7 cells incubated with TBTDC NPs was detected via CCK-8 assay. The results indicated that the cell survival rate of TBTDC NPs was about 11% after 10 min of white light irradiation (50 mW cm^−2^) at a relatively low concentration (12.5 μg mL^−1^), confirming the excellent potential of TBTDC NPs in PDT for cancer cell ablation. The PDT effect of TBTDC NPs was further investigated in MCF-7 tumor-bearing nude mice. According to the experimental results, the tumor growth of TBTDC NPs with irradiation group was significantly inhibited compared with other control groups after 14 days of treatment. Huang et al. also prepared single AIEgen nanoparticles (BTZPP-NPs) with high-efficiency PDT, which could selectively target lysosomes in living cells with high contrast and up to 14 days of tracking ability [195]. In addition, 63% anti-tumor inhibition rate was obtained in the 4 T1 tumor-xenograft model experiments.

### Colorectal cancer

Colorectal cancer (CRC) is the third leading cause of cancer death in both men and women, with an estimated 52,980 deaths in the United States in 2021 [196]. According to estimates, 10.5% of new colorectal cancer cases occur in people under the age of 50, which is increasingly younger [197]. Specifically, from 2000–2002 to 2014–2016, the incidence of colorectal cancer (specifically adenocarcinoma) in adults aged 40–49 has increased by nearly 15% [198]. Currently, there are many types of screening diagnostic methods for CRC, including guaiac fecal occult blood test (gFOBT), fecal immunochemical test (FIT) for hemoglobin, optical colonoscopy (OC) and flexible sigmoidoscopy (FS), etc. [199]. Surgery is the main treatment for localized disease, and combined with postoperative chemotherapy can reduce disease recurrence, especially if the disease has spread to the lymph nodes [200]. Radiation therapy, usually used in combination with chemotherapy before surgery, is one of the main treatments for CRC [201].

CRC fails to be diagnosed and treated early mainly because of the lack of specific/sensitive biomarkers for early detection. Shen and Tao et al. collaborated to report a mitochondrion-targeted TPE-IQ-2O with AIE property based the differential diagnosis of tumor and non-tumor cells in colorectal tissue, which lay the foundation for treatment of CRC [202]. Furthermore, due to the limited efficacy of conventional treatment, advanced CRC has poor prognosis with high risks of metastasis and recurrence. For the past few years, prominent progress has been made in the development of new treatments for diseases, among which PDT is a promising radical treatment for CRC [203]. Yang et al. reported that cationic luminescent progenitor TPE-DQN, with AIE properties, specifically stained mitochondria within cancer cells, helping them to differentiate cancer cells from normal cells [204]. Under white light irradiation, TPE-DQN could effectively ^1^O_2_ and the efficiency is up to 0.83. The advantages of PE-DQN lay the foundation for image-guided PDT to treat cancer cells and inhibit tumors. The observation in CT26-colon tumor-bearing mice further confirmed the therapeutic efficacy of TPE-DQN in vivo. For example, intratumoral injection of TPE-DQN followed by white light irradiation could entirely suppress tumor growth in mice after 10 days of treatments, while intratumoral injection of normal saline had no effect on tumor inhibition regardless of white light irradiation or under dark. TPE-DQN without light irradiation also showed no inhibition of tumor growth. Meanwhile, the body weight loss of each group was negligible during PDT. These phenomena were sufficient to indicate that TPE-DQN could be used as a highly biocompatible PS to inhibit tumor growth through PDT effect.

**Fig. 12:**
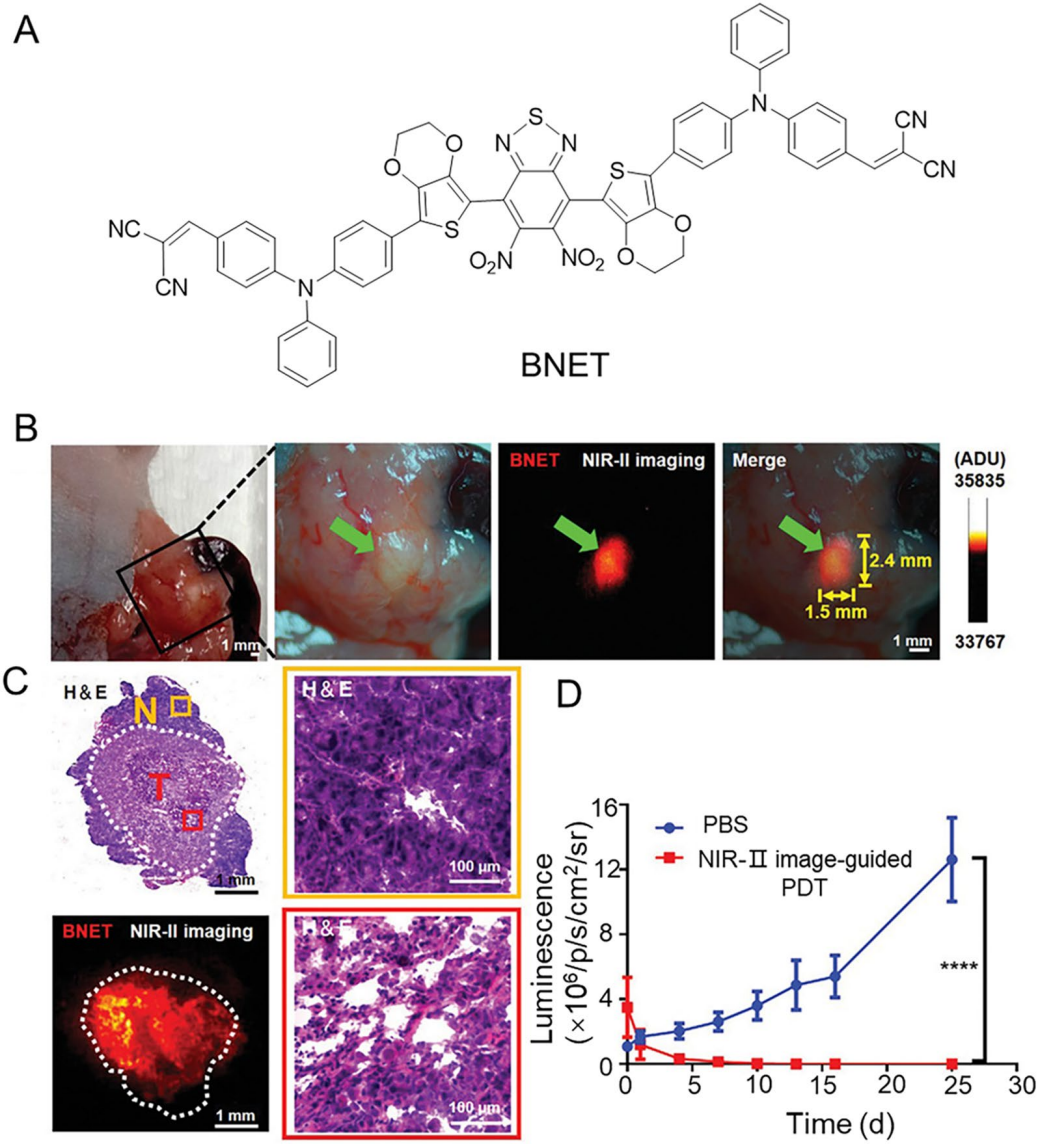
**A** The structure of BNET. **B** Intraoperative NIR-II imaging of nude mice bearing orthotopic Panc-1-luc pancreatic tumor by BNET NPs. **C** H&E staining and NIR-II real-time imaging of tumor sections. Dotted circles, areas of tumor. T, tumor; N, normal pancreatic tissue. **D** Tumor growth curves after different treatments were measured by the bioluminescence. Data are mean ± SD (n = 5). Significant statistical difference between the NIR-II image-guided PDT group and the PBS group (****p < 0.0001). Reproduced with permission [205]. Copyright 2021, Wiley–VCH

Activated by long wave light, PSs can minimize tissue decay and kill cancer cells without causing irreversible damage to normal cells. Therefore, the excitation wavelength of PSs for CRC treatment is mainly concentrated in the NIR, even reaches the NIR II. Gao et al. designed a series of PSs by adjusting the steric hindrance of molecules [205]. Among them, NIR-II PS nanoparticles prepared by BNET and albumin exhibited high-efficiency ^1^O_2_ generation, good photostability, as well as negligible dark toxicity. As shown in Fig. 12, The nanoparticles demonstrated highly specific NIR-II fluorescence imaging as well as efficient image-guided PDT in mice with in situ colon cancer or pancreatic cancer. The engineered nanoparticles revealed great potential in biomedical applications. In addition, the specific microenvironment of the tumor region becomes the trigger condition that can activate the PSs for CRC. For example, Min et al. reported a water-soluble AIE-PS with high targeting ability based on the acidic microenvironment of tumors [206]. Notably, the reversible control of _1_O^2^ production and photodynamic targeting of cancer cells in cultured and tumor-bearing mice in an acidic environment (pH 5.2) were permitted. Furthermore, the concentration of glutathione (GSH) in tumors is much higher than in normal tissues, which is considered to be a prerequisite for PS activation. Zhang et al. designed a novel ferrocene-modified vinyl pyridinium-substituted tetraphenyl ethylene (TPEPY-S-Fc) with disulfide bonds as an activated PS for GSH [207]. The fluorescence and _1_O^2^ generation of TPEPY-S-Fc were blocked in the presence of ferrocene. When GSH is encountered, the S–S bond breaks and the PDT-active Tpepy-SH species is released. Finally, TPEPY-SH can induce CT-26 cells apoptosis by generating _1_O^2^ under light irradiation.

### Prostate cancer

Prostate cancer is reported to be one of the main causes of death in men in the United States and the second most common type of cancer diagnosed in men worldwide [179, 208, 209]. Notably, the 5-year survival rates for patients with prostate cancer are variable and depend on clinical stage. According to the American Cancer Society (cancer.org), men with localized or regional prostate cancer had a 5-year survival rate of nearly 100%. However, for men diagnosed with distal metastasis, the survival rate is only 30%. Currently, localized prostate cancer is treated mainly with surgical removal of the gland or radiation therapy, which has been shown to be feasible [210]. However, for patients at high risk of distal metastasis, hormonal deprivation caused by surgical removal of the gland which leads to castration of resistant prostate cancer (CRPC) is a major clinical challenge [211]. Therefore, the development of medical tools and new treatment methods is of great significance for the timely diagnosis of the diseases.

**Fig. 13:**
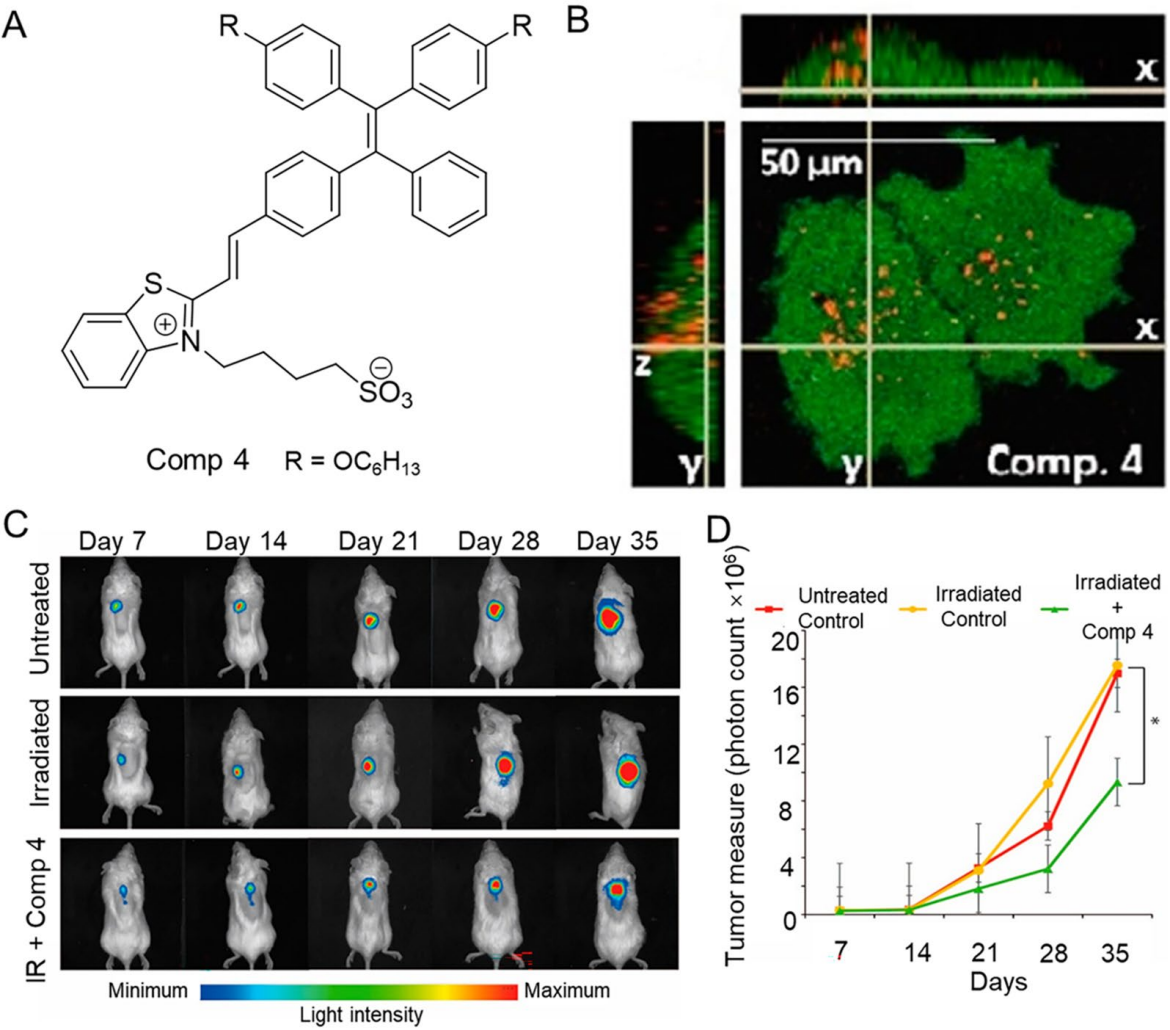
**A** The structure of conjugate 4. **B** Confocal laser scanning microscopy (CLSM) images of PC3 cell after an overnight incubation with TPE-NPs of conjugate 4, including orthogonal views, left and top rectangular panels. **C** Representative noninvasive bioluminescence images of different treatment groups implanted with PLuc-expressing PC-3 tumors s.c. in SCID mice. **D** Growth response of human PC-3 tumors to different treatment regimens. Reproduced with permission [213]. Copyright 2016, American Chemical Society

Local and metastatic recurrence of prostate cancer usually occurs after radical excision of the primary tumor is attempted. Therefore, during surgical excision of prostate cancer, technologies that can improve the visualization of tumor margins and adjunctive therapies to ablate remaining tumor tissue are needed. AIE-PSs possess the potential to combine fluorescence image-guided surgery with PDT to resect and ablate cancer cells [212]. Jayaram synthesized novel TPE conjugated compounds that underwent unique self-assembly to form spherical nanoparticles (TPE-NPs) with a size of 10 ± 5 nm, which displayed AIE emission in the near-infrared region [213]. As shown in Fig. 13, the potential use of TPE conjugated 4 as near-infrared fluorescent probes and PDT agents was investigated. Compared with normal cells, TPE-NPs showed an unusual preference for tumor cells and were localized in the cytoplasm. ROS produced by TPE conjugated 4 in PC3 cells under light irradiation were analyzed as one of the reasons for photoinduced cytotoxicity. Further evidence of the potential of PDT was obtained using immunodeficient SCID mice implanted subcutaneously with luciferase expressed human prostate tumor cells (PC3) on their backs. In short, the treatment regimen starts after 14 days of tumor cell growth. The mice were divided into 3 groups, in which the experimental group was given TPE conjugated 4 (50 μL, 10 μM) and blue (450 nm) LED lamp irradiation (20 W, 5 min) at the distance of 5 cm. In the other two groups, one group was irradiated at 450 nm for 5 min, while the other was untreated. PLuc activity of the PC3 tumors was monitored weekly by bioluminescence imaging (BLI) and the tumor sizes were measured, followed by continued treatment. The results showed that the tumor volume of the experimental group was observably smaller than that of the other two groups, and the bioluminescence intensity was reduced, indicating that PDT significantly reduced the tumor growth.

Although studies on the use of AIE photosensitizer in the treatment of prostate cancer are currently scarce, PDT therapy has shown promise in the treatment of primary and local prostate cancer after radiotherapy. Moreover, the characteristics of prostate cancer can also be used as a reference factor for the design of functional AIE-PSs, in which specific ligand is a very important part, such as FA [214], CD133 antibody [215], cation-independent mannose 6-phosphate receptor [216], prostate-specific membrane antigen (PSMA) [217]. We hope PDT can give full play to its own advantages and provide successful treatment in combination with the characteristics of prostate.

### Liver cancer

The liver is the body's largest and most important metabolic organ, which undertakes most of the body's toxin discharge. There are many possible causes of liver cancer, such as obesity, diabetes, fatty liver and so on [218]. Liver cancer is a very serious public health problem with an increasing incidence every year, with 841,000 incident cases and 782,000 mortalities worldwide in 2018 alone [219]. Hepatocellular carcinoma (HCC) accounts for nearly 90% of the primary liver cancer and is the most common type of liver cancer [220]. Currently, the treatment of HCC mainly depends on the diagnosis stage of patients, among which only surgical resection or liver transplantation is performed for patients with Barcelona Clinic Liver Cancer (BCLC) stage 0 or A [221]. In contrast, transarterial chemoembolization (TACE) or transarterial radioembolization (TARE) is recommended for patients with BCLC stage B and C [222]. BCLC stage D is referred to as terminal stage, and the current best treatment is symptomatic treatment with supportive care. With the development of technology, local thermal ablative methods, including microwave ablations and radiofrequency, have been regarded as alternative strategies for HCC treatment in an increasing number of cases [223–225]. However, these existing methods may lead to some adverse complications, such as liver decompensation, bile duct damage, extrahepatic organ injury, etc. [226].

Advanced liver cancer is almost incurable and causes a large number of deaths each year. Therefore, non-invasive long-term cancer cell tracing is an effective and important tool to understand the genesis, development, and evolution of cancer cells. Teng and Chen collaborated to develop the NIR AIE-active polymeric dots (TNZ2tPPI-Tat NPs), which showed ultra-long fluorescence biometric imaging (7 days) in MHCC97-H cells and maintained clear fluorescence signal after 5 generations in three hepatocytes (Lo2, MHCC97-H, Hep 3B) [227]. More importantly, according to in vivo fluorescence imaging, TNZ2tPPI-Tat NPs remained strong brightness within 26 days, which was superior to commercial fluorescent probes Cy5 and AF647, etc. AIE quantum dots not only have excellent performance in liver cancer cell tracking, but also provide potential for the cure of HCC with their effective PDT. TPVRT dots developed by Zhang and his collaborators not only showed excellent ROS production in vitro, but also demonstrated excellent performance of long-term tumor tracking and photodynamic ablation in vivo [228]. HepG2 tumor-bearing mice were randomly divided into four groups, including injection of saline, light irradiation, intratumoral injection of TPVTR dots and light irradiation after intratumoral injection of TPVTR dots. Different treatment regimens are initiated when the tumor volume reaches about 100 to 200 mm^3^. The efficacy of PDT up to 22 days in vivo was evaluated based on body weight and tumor size recorded every 2 days of nude mice. The normal saline group grew rapidly, and had no significant difference with other control groups. On the contrary, tumor growth in the light irradiation after injection of TPVTR dots group disappeared significantly after 21 days, confirming the effective PDT efficiency of TPVTR dots in vivo.

**Fig. 14:**
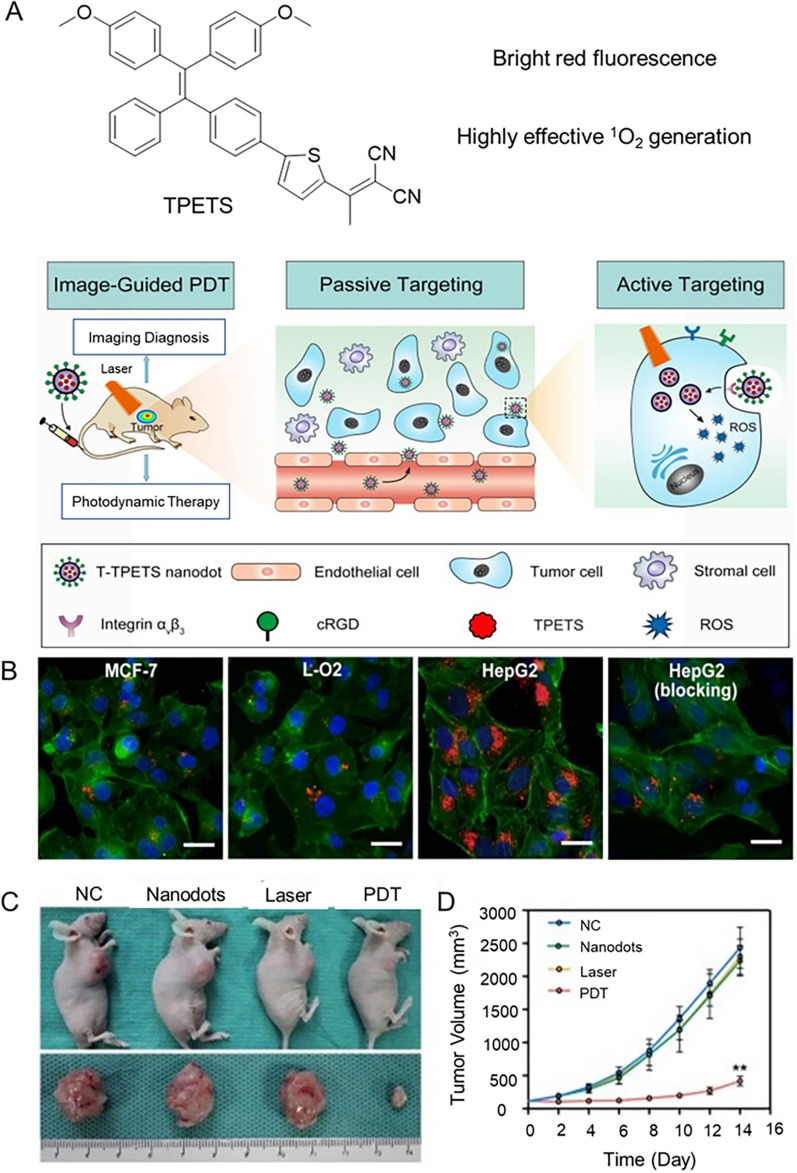
**A** The structure of TPETS and schematic illustration image-guided PDT mediated by T-TPETS nanodots in xenograft tumor model. **B** Confocal images of MCF-7, L-O2, and HepG2 cells after incubated with the T-TPETS nanodots. Red fluorescence: T-TPETS nanodots. Blue fluorescence: nuclei, green fluorescence: cytoskeleton. Scale bar: 20 μm. **C** Photographs of xenografts and tumors after different treatments. **D** Changes in tumor volume at different time points after treatment in the 4 groups (n = 3 per group). Data represent mean ± SD. Reproduced with permission [230]. Copyright 2019, Ivyspring International

Epithelial cell adhesion molecule (EpCAM) is overexpressed in HCC, so anti-EPCAM aptamer is used to functionalize nanoscale surfaces to specifically target liver cancer cells. Dineshkumar et al. fabricated mesoporous silica hollow nanospheres (MSHN) encapsulated by AIE-active polymer (PTPA), which were surface modified with anti-EPCAM aptamers to specifically target Huh-7 cells and were effectively internalized [229]. In addition, integrin α_v_β_3_ overexpressed in HCC cells is one of the targets. As shown in Fig. 14, Gao et al. designed and synthesized AIE organic nanodots (T-TPETS) targeting integrin α_ν_β_3_ for image-guided PDT [230]. We know that sub-organelles targeting HCC cells can also promote PDT therapy. Therefore, Zhao et al. developed two long-wavelength therapeutic probes (DCMT and DCMC) with AIE properties, which are modified with triphenyl-phosphonium cation to actively target the mitochondria of hepatoma cells [231]. HepG2 cells were taken as an example to evaluate the PDT efficiency of DCMT and DCMC. First, the cytotoxicity of probes for cells was assessed after incubation with the compound (0 to 40 μM) in the absence of light for 24 h, showing that the cells were viable. Of concern was that concentration-dependent cell death was observed when cells were irradiated with broadband light (22.7 mW cm^−2^, 100 min). After treatment with 40 μM DCMT and DCMC, cell viability was decreased by 74% and 61%, respectively. When compared with RB, the PDT effect of the probe was superior to that of RB, indicating that the synthetic probe was more prominent than that of the commercial reagent at relatively low concentrations and was capable to kill more HepG2 cells.

### Bladder cancer

Bladder cancer is the second most common genitourinary malignancy that originates in bladder cells. Most bladder cancers are diagnosed at an early stage, when they are highly treatable. From 2011 to 2017, the 5-year survival rate for carcinoma in situ of the bladder (by race and stage at diagnosis) was 96% for all ethnic groups in the United States [179]. But even early-stage bladder cancer can recur after successful treatment. For this reason, bladder cancer patients often require follow-up tests several years after treatment to detect a recurrence. As a result, bladder cancer has the highest cost from diagnosis to treatment.

**Fig. 15:**
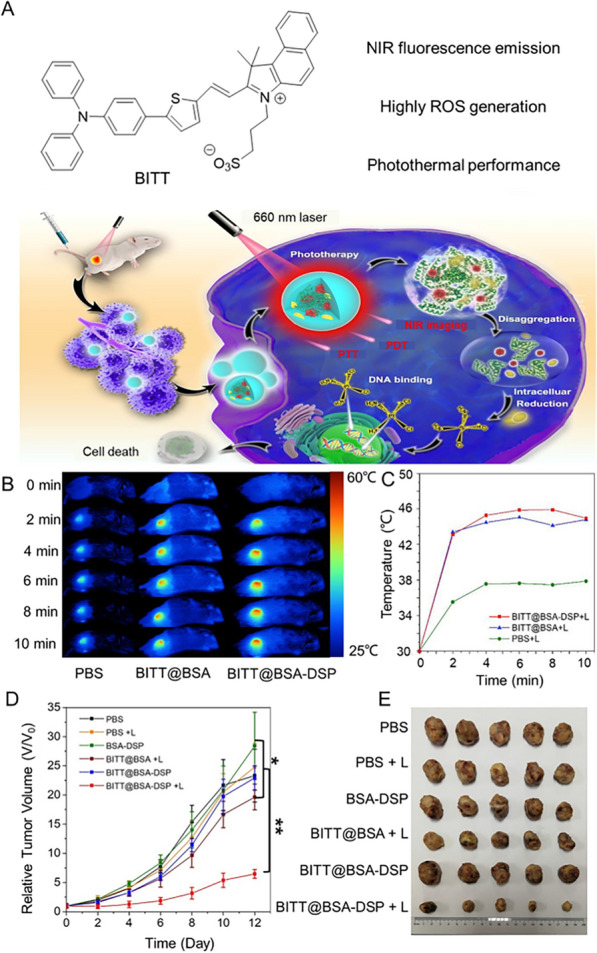
**A** The structure of BITT and schematic illustrations and the treatment of bladder cancer by NIR FLI-guided photo-enhanced chemotherapy. **B** Infrared thermography of bladder tumor mice 6 h after intravenous injection with PBS, BITT@BSA NPs, and BITT@BSA–DSP NPs, followed by laser irradiation (660 nm, 0.3 W cm^–2^, 10 min). **C** Average temperature of tumor regions. **D** Relative tumor volume growth curves of MB 49 tumor-bearing mice. **E** Representative tumor images of mice 12 days after different treatments. Reproduced with permission [232]. Copyright 2022, American Chemical Society

Currently, cisplatinum-based neoadjuvant chemotherapy (NAC) combined with surgical resection plays a key role in the treatment of bladder cancer, but its toxic and side effects severely limit its applications. Therefore, there is an urgent need to combine different treatment modalities with reduced drug dosages to defeat cancer. To solve this problem, Ding and Wang et al. developed a light-enhanced cancer chemotherapy (PECC) strategy based on multifunctional BITT@BSA-DSP NPs, which had excellent PDT and PTT [232]. At the same time, BITT@BSA-DSP NPs could be effectively taken up by bladder cancer cells and then release Pt (II) under the action of reductase, ensuring the efficacy of chemotherapy (Fig. 15). Most importantly, the study of PECC in vivo in MB49 tumor-bearing mice showed that the BITT@BSA-DSP + laser group had a significant tumor suppressive effect compared with other groups, indicating that PECC has an obvious tumor inhibitory of treatment. In addition, there was no significant difference in body weight growth curve among all groups during treatment. These results confirmed that PECC integrated with NIR FL-guided imaging effectively promoted sensitivity of bladder cancer to cisplatin chemotherapy with minimal side effects. This work provides a promising strategy for improving the sensitivity of multiple of cancer to chemotherapeutic drugs and even for effective treatment of drug resistance. In addition, sonodynamic therapy (SDT) is regarded as an effective method for cancer treatment due to its advantages of deep tumor penetration and high therapeutic efficacy. Guo et al. utilized a patient-derived MVs/AIEgen hybrid system (AMVs) for personalized SDT in a patient-derived xenograft (PDX) model of bladder cancer [233]. Impressive AMVs on PDX models displayed excellent tumor targeting capability and effective personalized SDT therapy.

In recent years, targeting molecules against bladder cancer has also been one of the focus of researchers. He et al. encapsulated AIE molecules with PEG and modified them with RGD on the surface to prepare a dual-function TPE-red-PEG-RGD nanoparticles that could be applied to target bladder cancer and image-guided PDT in vivo [234]. A combination of passive and active targeting was used to ensure the therapeutic effect, and targeted imaging in tumor regions and high anti-tumor efficacy could be achieved at a reasonable low dose (10 mg kg^−1^). Besides, the combination of inorganic nanomaterials and AIE-PSs shows great potential to enhance the efficiency of PDT. They also modified nanographene oxides (NGO) with PEG chains and attempted to evaluate in vitro and in vivo PDT effects using NGP coated AIE nanoparticles (NGP-TPEred) [235]. PDT was evaluated using the UMUC3 (human bladder cancer cell line) tumor xenograft model. Tumor growth was immediately completely inhibited with the presence of NGP-TPEred nanoparticles and 450 nm laser irradiation, showing a significant difference compared to the other three groups. This shows the potential for NGP-TPEred nanoparticles to be used in PDT of bladder cancer.

PDT plays a particularly crucial role in cancer treatment. However, PDT remains poor due to the insufficient production of PS by ROS and the aggravation of hypoxia in the tumor microenvironment. To solve this problem, Guo et al. used the previously reported TFM as a ROS producer, together with the oxygen regulator doxycycline (DOXY), both of which are encapsulated into the NPs (D–P–T–D NPs), by utilizing powerful PDT in the oxygen-enriched tumor microenvironment and inhibiting tumor migration via regulating hypoxic-related pathways to defeat tumors [236]. Specifically, D–P–T–D NPs act on the tumor site by an EPR effect, and TFM is released from the NPs to produce powerful ROS with a 650 nm laser irradiation. With the introduction of DOXY, mitochondrial respiration was suppressed to overcome hypoxia by reducing endogenous oxygen consumption, further enhancing the efficacy of TFM-mediated PDT. Subsequent in vivo experiments verified that D–P–T–D NPs accumulated in tumor tissues through EPR effect, and reached a maximum accumulation in tumor tissues 4 h after injection to meet the therapeutic needs. In addition, D–P–T–D NPs exhibited excellent tumor killing effect under 650 nm laser irradiation (0.2 W, 5 min). Notably, there was almost no tumor growth in the D–P–T–D + Laser group during treatment, and more importantly, D–P–T–D NPs can inhibit tumor migration after PDT, thus improving the PDT effect. In short, the novel D–P–T–D NPs combined the optimized PDT effect and the inhibitory effect of tumor migration after PDT, suggesting a potent antitumor effect in the treatment of advanced bladder cancer.

### Cholangiocarcinoma

Cholangiocarcinoma (CCA) is a malignant tumor originating in the extrahepatic bile duct from the hilar region to the lower common bile duct [237]. The incidence of CCA has increased significantly in the past few decades [238]. Surgical resection as the primary treatment for early cases is a good candidate for treatment of all subtypes of CCA [239]. However, the onset of CCA is insidious and the early symptoms are not obvious, so most patients are diagnosed at an advanced stage, which leads to the easy loss of the opportunity of surgical resection [240]. CCA is not sensitive to radiotherapy or chemotherapy in most cases, including gemcitabine and cisplatin chemotherapy, resulting in unsatisfactory efficacy and poor prognosis [241]. In order to improve the long-term survival probability of CCA patients, it is urgent to develop alternative treatment strategies in addition to surgical treatment [242, 243]. Since the first successful case of PDT in unresectable CCA was revealed, many clinical trials have indicated that PDT combined with biliary decompression can improve survival and post-treatment quality of life for CCA patients over the years [244–246].

**Fig. 16:**
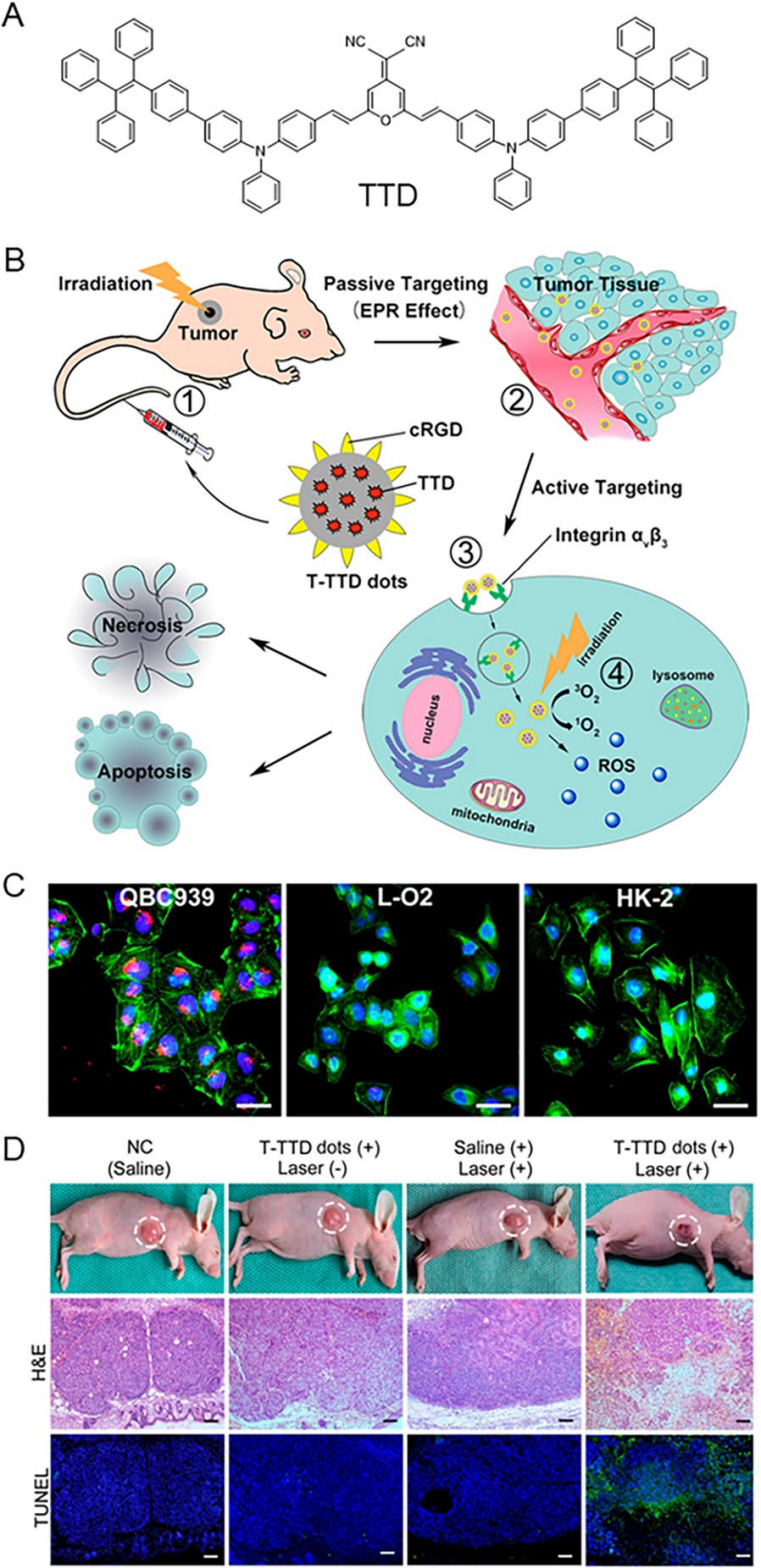
**A** The structure of T-TTD. **B** Schematic illustration of T-TTD dots-mediated PDT in a human cholangiocarcinoma xenograft mouse model. **C** Fluorescent images of different cells after incubation with T-TTD dots for 4 h. Red fluorescence: T-TTD dots. Blue fluorescence: nuclei. Green fluorescence: cytoskeleton. Scale bars: 50 μm. **D** Photograph of a xenograft tumor 3 days after different treatments (white circles). H&E staining for pathological changes and TUNEL staining for apoptosis (green) in tumor sections. Scale:100 μm. Reproduced with permission [248]. Copyright 2017, American Chemical Society

Most patients with extrahepatic CCA are diagnosed as advanced stage and therefore cannot undergo timely curative surgical resection [247]. PDT is a highly effective ablative method of cancer cells, and has emerged as an alternative treatment strategy to increase the probability of long-term survival in patients with unresectable extrahepatic CCA. In order to achieve the broad application value of AIE materials in clinical diagnosis and treatment of CCA, Zheng and Liu et al. collaborated to develop an ideal PS (TTD) with long absorption wavelength to ensure the effect of photodynamic therapy on CCA [248]. As shown in Fig. 16, targeted delivery of NPs has been used to improve material enrichment at the tumor, so they take advantage of TTD to fabricate organic AIE dots with surface modification cRGD for image-guided PDT. Specifically, specific interaction between integrin α_ν_β_3_ and cRGD on T-TTD dots promoted the site to target tumor cells. To prove the above idea, the uptake of T-TTD dots in different cell lines, including QBC939 cells overexpressing α_ν_β_3_, normal L-O2 and HK-2 cells has been studied by CLSM and flow cytometry. All the experimental results were sufficient to prove that the integrin α_ν_β_3_ receptor-mediated endocytosis promoted the uptake of T-TTD dots. Next, synthetic T-TTD dots were injected into tumor-bearing mice to further evaluate the treatment effect of T-TTD dots-mediated by PDT in vivo, which could accumulate into tumor interstitial fluid through enhanced permeability and EPR effects. Tumor-bearing mice were divided into four groups, one of which was injected with T-TTD dots followed by laser irradiation (530 nm, 250 mW cm^−2^), the tumor size decreased significantly, in contrast, tumors in the other three groups grew rapidly, including the group of saline injection only via the tail vein, the group of saline injection followed by laser irradiation (530 nm, 250 mW cm^−2^) and T-TTD dots injection without laser irradiation. These results suggest that T-TTD dots-mediated PDT is an effective and desirable treatment for CCA ablation. This makes AIE materials more promising for translational studies in the diagnosis and treatment of CCA in the future.

Study confirms that it is feasible to effectively ablate extrahepatic CCA cells through mitochondrial injury pathway. Li and Gao et al. are committed to developing PSs for PDT of CCA [249]. A mitochondrial anchored PS, TTVPHE, developed to effectively ablate extracellular CCA cells through mitochondrial injury pathway. When the irradiation time was extended to 15 min or the concentration of TTVPHE was increased to 7 mM, the extrahepatic CCA QBC939 cells viability was almost zero, indicating that TTVPHE had sufficient ability to kill cells and lay a foundation for tumor elimination in vivo.

## Summary and outlook

PDT, as a novel non-invasive treatment method, has attracted extensive attention in biomedical applications. PS is one of the essential components of PDT, so its ROS production level directly determines the effect of PDT. It also has the advantage of fluorescence to combine optical imaging with PDT. This article reviews the current strategies of PDT in various tumors and guidelines for the development of high-efficiency AIE PSs. However, there are still many limitations in the design of efficient AIE PSs, and the PDT strategies based on AIE-PSs are still in their infancy, which requires continuous efforts to be applied in clinical practice. Here, we present some of the challenges of AIE PSs in tumor PDT treatment, and propose important research directions that require joint efforts.

1. The efficient activation of PSs by light sources that can penetrate deep into biological tissues plays an important role in optical PDT. However, the excitation peaks of most AIE PSs are the same as those of clinically approved PSs (*e.g.*, porphyrins and their derivatives), usually in the visible light range, resulting in shallow tissue penetration, which seriously impede their clinical application. Near infrared AIE PSs with highly ROS generation capability is developed to improve tissue penetration depth, such as two-photon photosensitizer. The emission wavelength of some PSs is covered by the background fluorescence of organisms, which is an urgent problem to be solved. So far, the reported AIE-PSs usually show emission peaks in the range of 600–700 nm, leading to problems such as low tissue imaging sensitivity. Therefore, highly efficient AIE-PSs with long-wavelength absorption and emission are highly desirable to advance improve the treatment accuracy and achieve visualized phototheranostics of cancer.
2. To overcome the toxicity and lack of specificity of photosensitizer and improve its bioavailability is always a problem to be solved. Nanomaterials have been widely modified to treat cancer. Although the number of studies has been increasing, the approved nanodrugs have not increased much in recent years. In order to better improve clinical transformation, further research is needed for targeted delivery of nanocarriers to reduce toxicity, enhance permeability and retention and avoid immune system clearance.
3. The cytotoxicity mechanism of PDT is different from the ones of chemotherapy, radiation therapy, immunotherapy and so on, so it presents unique advantages in treatment. PDT effects may be at least partially localized to the tumor, leading to cell death within tumors and reducing damage to normal cells. Moreover, PDT uses non-ionizing radiation in most cases, potentially promoting treated volume healing after treatment. To our pleasure, PDT can be used as many times as the clinician requires, which is not possible with currently available treatments such as surgery, chemotherapy, and radiation. In particular, PDT unique therapeutic mechanisms can inhibit drug resistance pathways and re-sensitize resistant cells to standard therapies. Despite the great potential of PDT, its application to deep-seated cancers and metastases remains challenging. It is mainly manifested in limited light penetration depth, non-ideal PSs, and complicated implementations in the clinic. Single PDT has inevitable drawbacks, so combining other therapies to effectively treat tumors is one of the desirable strategies. These therapies mainly include surgical treatment, chemotherapy, immunotherapy, radiotherapy, protein therapy, gene therapy and so on. Meanwhile, the synergistic effect of AIE PSs combined multiple treatments can be further enhanced via developing the functional design of material system.
4. Accurate and sensitive diagnosis and visual guidance are considered necessary to achieve accurate and efficient PDT. Conventional single-mode imaging methods have their own inherent defects. Fortunately, multi-mode imaging can complement each other to achieve the desired goal. Multifunctional phototherapy materials were exploited undoubtedly of great value for cancer treatment.
5. PDT is based on the phototoxicity of PSs after irradiation. However, most clinically approved PSs will be widely distributed in normal tissues, particularly in the skin, where they can produce phototoxicity with serious side effects on exposure to light. As a result, the patients have to stay in a dark room for hours or even weeks during or after a PDT. AIE PSs designed to be sensitized in specific environments or conditions has been identified as a simple and effective way to liberate patients, which deserves further study.

At present, PDT has revealed unique advantages especially in the field of malignant tumors and skin diseases, especially in lung cancer, prostate cancer and skin tumor. The basic research and clinical application of PDT are in an active development trend. With the development of PSs from the first to the third generation, PDT has correspondingly more favorable properties, which can be better used in clinical trials. In this article, we put forward the advantages, limitations and future development direction of PDT based on AIE PSs in the field of tumor therapy. Encouragingly, there have been systemic and multifaceted demonstration using AIEgens for an optical imaging-guided surgical operation from small animals (mice and rabbits) to the typical non-human primate animal model (rhesus macaque). Although AIE PSs are still in their infancy for PDT of cancer, people have been making efforts and innovations in the mechanism of action and application in cancer treatment. The application limitations of PDT mentioned above are urgent problems for us to solve in the future. We hope to pay attention to current research hotspots and promote the further development of AIE PSs design, so that it can give full play to its advantages in clinical application.

## Data Availability

Not applicable.

## Abbreviations

PDT: Photodynamic therapy
PSs: Photosensitizers
ACQ: Aggregation-caused quenching
ROS: Reactive oxygen species
AIE: Aggregation-induced emission
FDA: Food and Drug Administration
HPD: Porfimer sodium (Photofrin)
ALA: 5-Aminolevulinic acid
mTHPC: Temoporfin (Foscan)
AIEgens: AIE luminogens
LAPTM4B: Lysosomal protein transmembrane 4 beta
PTT: Photothermal therapy
S_0_: The ground state
S_*n*_: Higher-energy orbitals
S_1_: The lowest singlet excited state
IC: Internal conversion
ISC: Intersystem crossover
ΔE_ST_: The energy gap between excited S_1_ and T_1_ states
RIM: Restricted intermolecular motion
D: Electron donor
A: Electron acceptor
HOMO: The highest occupied molecular orbital
LUMO: The lowest unoccupied molecular orbital
TPE: Tetraphenylethylene
BT: 2,1,3-Benzothiadiazole
TCF: 2-Dicyanomethylene-3-cyano-4,5,5-trimethyl-2,5-dihydrofuran
C = O: Carbonyl group
–CN: Cyanide group
SOC: Spin–orbit coupling
TPA: Triphenylamine
RB: Rose Bengal
ABDA: 9,10-Anthracenediyl-bis(methylene)dimalonic acid
SOSG: Singlet Oxygen Sensor Green
PQ: 9,10-Phenanthrenequinone
AI-ISC: Aggregation-induced intersystem crossover
FA: Folic acid
PEG: Polyethylene glycol
PICAL: Photo-immune-regulating-liposome
BPD: Benzoporphyrin acid A
EGFR: Epidermal growth factor receptor
PPL: Preformed Plain Liposome
MPD: 1,2-Distearoyl-sn-glycero-3-phosphoethanolamine-N-[maleimide(polyethylene glycol)-2000]
R: Arginine
ER: Endoplasmic reticulum
ICD: Immunogenic cell death
hrHPV: High-risk human papillomavirus
CIN: Cervical intraepithelial neoplasia
HDACs: Histone deacetylases
GBM: Glioblastoma multiform
MDR: Multidrug resistance
BBB: Blood–brain barrier
BTB: Blood-tumor barrier
ApoE: Apolipoprotein E peptide
NIR-IIb: Near-infrared IIb
NK: Natural killer cells
MM: Malignant melanoma
NMSC: Non-melanoma skin cancer
CMM: Cutaneous malignant melanoma
NPs: Nanoparticles
BCC: Basal cell carcinoma
SCC: Squamous cell carcinoma
NSCLC: Non-small cell lung cancer
LDLR: Low-density lipoprotein receptors
LDL: Low-density lipoprotein
cis-Pt: Cisplatin
MCTSs: Multicellular tumor spheroids
Pt: Platinum
TPP: Triphenylphosphorus
FLI: Fluorescence imaging
PAI: Photoacoustic imaging
PTI: Photothermal imaging
HER2: Human epidermal growth factor receptor 2
CRC: Colorectal cancer
gFOBT: Guaiac fecal occult blood test
FIT: Fecal immunochemical test
FS: Flexible sigmoidoscopy
OC: Optical colonoscopy
GSH: Glutathione
CRPC: Castration of resistant prostate cancer
BLI: Bioluminescence imaging
PSMA: Prostate-specific membrane antigen
HCC: Hepatocellular carcinoma
BCLC: Barcelona Clinic Liver Cancer
TACE: Transarterial chemoembolization
TARE: Transarterial radioembolization
EpCAM: Epithelial cell adhesion molecule
MSHN: Mesoporous silica hollow nanospheres
NAC: Neoadjuvant chemotherapy
PECC: Photoenhanced cancer chemotherapy
SDT: Sonodynamic therapy
PDX: Patient-derived xenograft
NGO: Nanographene oxides
DOXY: Doxycycline
CCA: Cholangiocarcinoma
CLSM: Confocal laser scanning microscopy
